# *Panthera tigris* jacksoni Population Crash and Impending Extinction due to Environmental Perturbation and Human-Wildlife Conflict

**DOI:** 10.3390/ani11041032

**Published:** 2021-04-06

**Authors:** Dennis Choon Yung Ten, Rohana Jani, Noor Hashida Hashim, Salman Saaban, Abdul Kadir Abu Hashim, Mohd Tajuddin Abdullah

**Affiliations:** 1 Department of Wildlife and National Parks Pahang, Jalan Kompleks Tun Razak, Bandar Indera Mahkota, Kuantan 25582, Malaysia; 2 Institute for Advanced Studies, University of Malaya, Jalan Profesor Diraja Ungku Aziz, Kuala Lumpur 50603, Malaysia; 3 Faculty of Economics and Administration, University of Malaya, Jalan Profesor Diraja Ungku Aziz, Kuala Lumpur 50603, Malaysia; 4 Ungku Aziz Centre for Development Studies, University of Malaya, Jalan Profesor Diraja Ungku Aziz, Kuala Lumpur 50603, Malaysia; 5 Center for Foundation Studies in Science, University of Malaya, Jalan Profesor Diraja Ungku Aziz, Kuala Lumpur 50603, Malaysia; nhhpasum@um.edu.my; 6 Department of Wildlife and National Parks Johor, Blok B, Wisma Persekutuan, 9th Floor, Jalan Air Molek, Johor Bahru 80000, Malaysia; salman@wildlife.gov.my; 7 Department of Wildlife and National Parks, KM10, Jalan Cheras, Kuala Lumpur 56100, Malaysia; kadir@wildlife.gov.my; 8 Institute of Tropical Biodiversity and Sustainable Development, Universiti Malaysia Terengganu, Kuala Nerus 21030, Malaysia; 9 Academy of Sciences Malaysia, West Wing, MATRADE Tower, Level 20, Jalan Sultan Haji Ahmad Shah, Kuala Lumpur 50480, Malaysia

**Keywords:** wildlife management, wildlife strategies, anthropogenic disturbances, environmental perturbation, infectious diseases

## Abstract

**Simple Summary:**

The Malayan tiger, with less than 200 individuals in Malaysia, is in an intermediate population crash. Anthropogenic disturbances (poaching, roadkill, and human–tiger conflict), environmental perturbation (decreasing habitat quality), and infectious diseases have been identified as factors leading to impending extinction. Preliminary findings from stakeholders indicate Peninsular Malaysia has an existing Malayan Tiger conservation management programme. However, to enhance the protection and conservation of the Malayan Tiger, the authority should re-assess the existing legislation, regulation, and management plan, and realign them to prevent population decline.

**Abstract:**

The critically endangered Malayan tiger (*Panthera tigris* jacksoni), with an estimated population of less than 200 individuals left in isolated rainforest habitats in Malaysia, is in an intermediate population crash leading to extinction in the next decade. The population has decreased significantly by illegal poaching, environmental perturbation, roadkill, and being captured during human–wildlife conflicts. Forty-five or more individuals were extracted from the wild (four animals captured due to conflict, one death due to canine distemper, one roadkilled, and 39 poached) in the 12 years between 2008–2019. The Malayan tigers are the first wildlife species to test positive for COVID-19 and are subject to the Canine Distemper Virus. These anthropogenic disturbances (poaching and human–tiger conflict) and environmental perturbation (decreasing habitat coverage and quality) have long been identified as impending extinction factors. Roadkill and infectious diseases have emerged recently as new confounding factors threatening Malayan tiger extinction in the near future. Peninsular Malaysia has an existing Malayan tiger conservation management plan; however, to enhance the protection and conservation of Malayan tigers from potential extinction, the authority should reassess the existing legislation, regulation, and management plan and realign them to prevent further population decline, and to better enable preparedness and readiness for the ongoing pandemic and future threats.

## 1. Introduction

The critically endangered Malayan tiger (*Panthera tigris* jacksoni) is in an intermediate population crash leading to extinction in the wild by the next decade. It is estimated that less than 200 individuals are left in fragmented and isolated rainforest habitats in Malaysia.

The Malayan tiger is a large carnivore that plays an important ecological role by maintaining the balance between the interactions of predators, herbivores, and plant diversity for the stability of the rainforest ecosystems. The species is proudly displayed on the coat of arms of Malaysia as the symbol of strength and royal power in Malaysia [1]. Culturally, the folk-tales of the mighty Malayan tiger can be traced back centuries in the Malay culture. The myths have been written in articles such as some Malay Studies in the Journal of the Malayan Branch of the Royal Asiatic Society [2], the Malay Annals rewrite by Tun Sri Lanang in 1612 [3,4], and the Alfred Russel Wallace travelogue book entitled—The Malay Archipelago in 1869 in Melaka [5].

The Malayan tiger population is now facing severe and apparent threats of extinction. The Malayan tigers are threatened by anthropogenic disturbances (poaching, industrial agriculture expansion, commercial logging, and human settlement), environmental perturbation (disturbances, decreasing habitat quality, and pollution), trade in traditional Chinese medicine using illegal tiger products [6,7,8], and by diseases [8,9,10,11,12].

Through the Department of Wildlife and National Parks (DWNP), Malaysia’s Government has always placed top priority on Malayan tiger conservation. The DWNP recognised the Malayan tiger as a totally protected species and a critically endangered species, as published in the Malaysian mammals red list in 2017 [13].

Regarding species conservation, Malaysia has started systematically implementing the national conservation initiative–the Malaysia National Tiger Conservation Action Plan (NTCAP) in 2009 with the intended goal to double the country’s tiger population from then 500 to 1000 by the year 2020 [1]. The NTCAP has drafted a series of specific actions to aid the wild tiger population in Malaysia, focussing on habitat protection, species protection, human–tiger conflict, and research. However, the NTCAP focusses on species in situ conservation but with minimal attention to the species’ ex situ conservation. Considering the latest population count of wild Malayan tigers in 2018, the conservation actions of the NTCAP have diverted from the original goals. During 2018, the Malaysian Government launched the major “Save the Malayan Tiger” campaign. The campaign has employed an integrated approach. There are five programmes: conservation, research, funding and corporate social responsibility, enforcement, awareness publicity and promotion campaigns. The new Malayan tiger conservation planning has included ex situ conservation. It aims to establish the National Tiger Conservation Center and venture into Malayan tiger captive breeding under conservation and research programme.

Following the launch of the NTCAP in 2009, the Government of Malaysia, through the Department of Town and Country Planning (DTCP), followed up by establishing the Central Forest Spine (CFS): Master Plan for Ecological Linkages in 2010, the main objective of which is to create ecological linkages for environmentally sensitive areas. NTCAP has been used as the main reference to stimulate the establishment of the linkages. Meanwhile, the government also began the “Save the Malayan Tiger” campaign in 2018 for integrated conservation management planning, including the ex situ dimension. However, as Sanderson, et al. [14] mentioned, sometimes good science is necessary but not sufficient for conservation. Successful tiger conservation needs support from the tiger community stakeholder. The government and the non-government organisations need to support and integrate the conservation research findings into development plans and the people’s daily consciousness [14]. The NTCAP and the “Save the Malayan Tiger” campaign are the result of integrated and holistic tiger conservation efforts. However, the wild population downward trend is persisting. The population figures indicate the Malayan tiger population has lost a big portion of its natural population, with approximately 200 wild individuals now [15,16] and 65 captive individuals in Malaysia [12]. The Malayan tiger population in Malaysia is critically threatened [6], and the wild Malayan tigers potentially will collapse [17] by 2022, as predicted by the World Wildlife Fund (WWF)-Malaysia [18]. Looking at the current wild population status, we are close to losing the whole population across the entire state range.

Therefore, this paper aims to present the tiger’s current conservation status and examine the tiger’s likeliness for impending extinction while impacted by anthropogenic disturbances due to environmental perturbation and recent zoonotic diseases.

## 2. Materials and Methods

We screened documents and internal annual reports from the Department of Wildlife and National Parks (DWNP) Malaysia. We conducted and analysed questionnaires from selected captive tiger facilities that housed captive Malayan tigers.

Based on available datasets, we interpreted the anthropogenic activities of the tropical lowland habitats, the impact of human–tiger conflict, and the roles of captive breeding to provide new insights for future global strategies for the management and perpetual conservation of this majestic critically endangered species for our next generation.

### 2.1. Sampling

The questionnaire was carried out from December 2018 to June 2019. During the present study, we distributed 73 questionnaires through emails or handed the hardcopy to the potential respondents with various positions in the 48 known captive tiger facilities globally and ten captive facilities in Malaysia that owned Malayan tigers. The zoos’ records consist of the Malayan tiger captive population based on datasets from the Department of Wildlife and National Parks (DWNP) Malaysia and the Malayan tiger (*Panthera tigris* jacksoni) Studbook which were extracted for this study [19].

The questionnaire distribution first was done using a pilot study in July 2018, where all the respondents’ unanswerable items were restructured. The questionnaire was registered under the University of Malaya and compiled according to the rules and regulations set under the University of Malaya Research Ethics Committee (reference number UM.TNC 2/UMREC -239).

#### 2.1.1. Questionnaire Development

The type of sampling approach applied was a cross-sectional sampling survey and involved data collection at one point in time [20]. This study involved a non-probability sampling method. The respondents were the directors, veterinarians, senior supervisors, and senior keepers from the captive animal institutions dealing with captive Malayan tiger ex situ management.

The instrument was developed using both “closed-ended” and “open-ended” techniques using the 1–5 Likert scale to allow the respondents to give a value for their opinions. The questionnaire was written in English and divided into five sections (Supplementary File 1).

Section A contained the respondent’s background with six questions about demographic details such as name, gender, education level, the field of expertise, the subfield of expertise, and the number of working years in the conservation field.

Section B contained the institution’s background. This Section had five questions: name of the institution, the number of wildlife species kept in the institution, institution category, and the respondents’ position in the institution.

Section C regarded animal captive management, and the questionnaire focussed on the origin and background of the Malayan tiger. The items regarded the animal population’s initial sources, the number of years the institution had housed the animals, and the purpose of the institution’s captive programme. Regarding the 89 questions in section C, the items were specifically designed to collect information and opinions on the species’ captive animal management. Most of the questions were close-ended questions except for two open-ended questions. The open-ended questions enabled the respondents to give their opinions on any other domains and items essential for the animal species and captive conservation management.

Section D regarded animal ex situ management and contained 33 questions to gather expert opinions on animal ex situ management. Regarding the ex situ conservation efforts for the species, the author also provided 15 questions to explore the estimated amount of cost to the institution for the particular animal species. This section also had one open-ended question to enable the respondents to list any other domains in which their opinions were important in the ex situ species management.

Last, section E was for general comments; five open-ended questions inquired about the respondents’ general comments and views regarding animal conservation management.

The questionnaire was designed in the simplest and most informative way to avoid misunderstandings, confusion, or bias amongst respondents. Some of the questions needed the respondents to give a rating based on the Likert 1–5 scale. The Likert scale required the respondents to agree or disagree with the statements on a five-point scale [21]. Considering the purpose of this research, we used four types of Likert scale for important (1 = very unimportant, 2 = unimportant, 3 = neither unimportant nor important, 4 = important and 5 = very important), agreement (1 = strongly disagree, 2 = disagree, 3 = neither agree nor disagree, 4 = agree and 5 = strongly agree), quality (1 = poor, 2 = fair, 3 = good, 4 = very good and 5 = excellent) and frequency (1 = never, 2 = rarely, 3 = occasionally, 4 = often and 5 = always).

#### 2.1.2. Quanlitative Data Analysis

The data were coded and analysed using the Statistical Package for Social Science software programme (IBM SPSS Statistic Version 20, 2011, Armonk, NY, USA, accessed on 15 September 2019). The percentage was used in representing the data (Supplementary File 2).

### 2.2. Secondary Data

We also compiled the Department of Wildlife and National Parks (DWNP’s) annual reports, published reports, and wildlife conflict database from 2008 to 2019 and classified them into various categories.

## 3. Results

The structured questionnaire was collected from 30 expert respondents (14 Malayan tiger captive facilities), including 20 local zoo respondents from five facilities, regarding the current governance of the ex situ Malayan tiger management. Malaysia has ten facilities that own captive Malayan tigers, with eight in Peninsular Malaysia and one each in the States of Sabah and Sarawak. We received 30 completed (41%) questionnaires (*n* = 30) from 14 captive animal facilities (29%) from 30 expert respondents. Out of the 14 facilities that gave a response, five were from Malaysia’s local captive animal facilities.

The 30 expert respondents were from zoos (26 respondents) and rescue centers (4 respondents) comprising executive-level (4), administrative-level (5), veterinarian or husbandry curator (13), and supervisor (8) responsibilities. Sixteen percent of the respondents were Masters degree holders, 50% were first degree holders, 17% were diploma holders, and certificate holders were 17%.

### 3.1. The Malayan Tiger Ex Situ Conservation Policy

**Figure 1:**
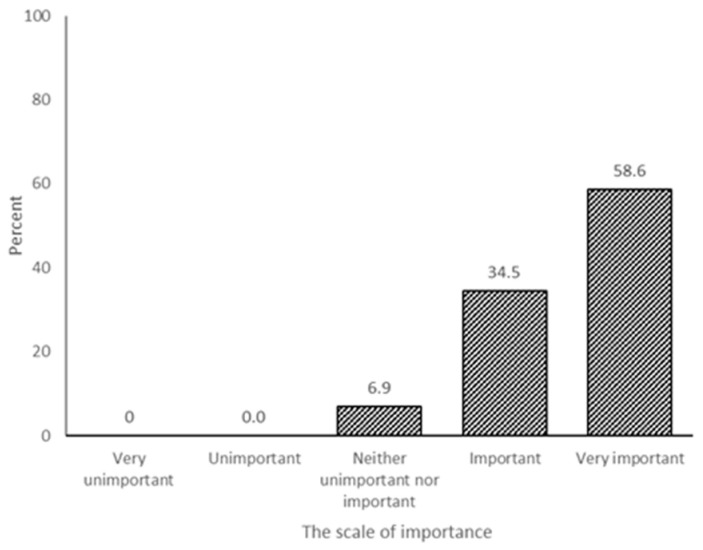
The importance of the integration of scientific knowledge in ex situ species management into the species’ conservation policy and practice.

**Figure 2:**
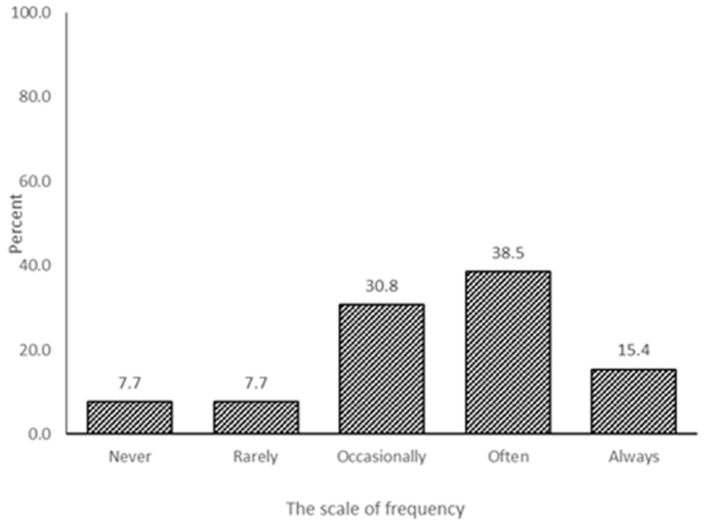
The existing integration of scientific knowledge of the captive species management into the species’ conservation policy and practice.

About 93% of the expert respondents agreed that it was important that scientific knowledge be integrated into the Malayan tiger conservation policy and practice (Figure 1). However, the expert respondents also noted that the current integration of scientific knowledge of Malayan tigers in species conservation policy and practice is 30.8% occasionally, 38.5% often, and 15.4% always (Figure 2).

**Figure 3:**
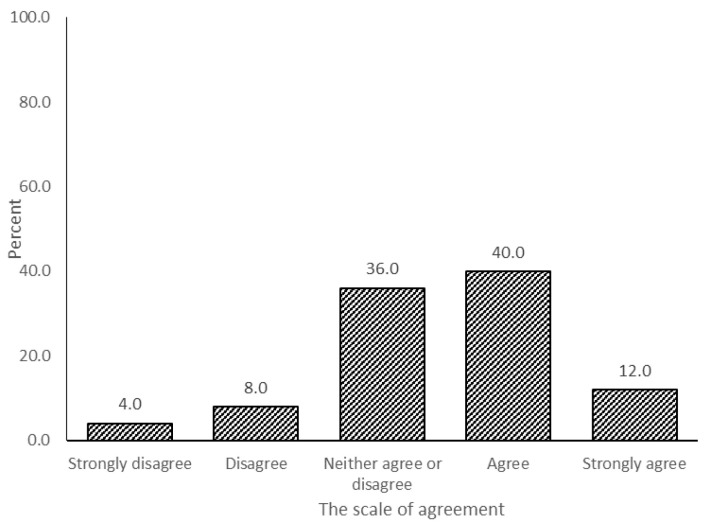
The implementation of the species strategic management plan and research procedures by the institution.

**Figure 4:**
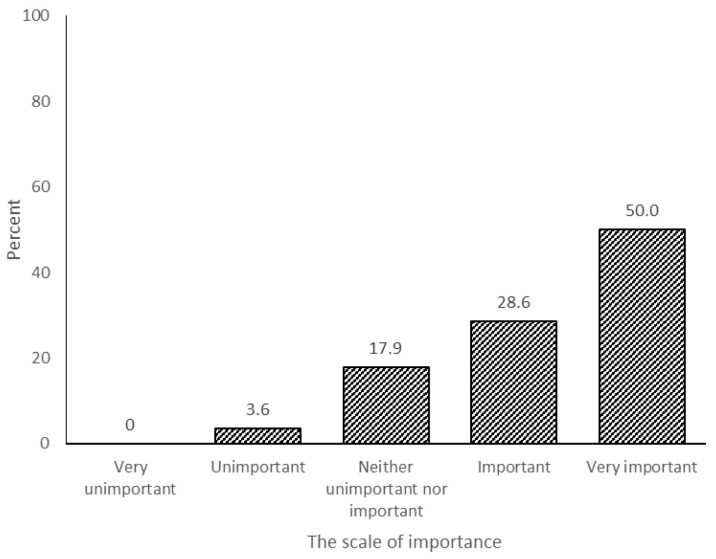
The need for a conservation policy or strategy or a species management plan by the captive animal institution from the animal host country.

Considering the survey, 52% of the respondents agreed that the institution must implement Malayan tiger strategic and management plans (Figure 3). To implement the plans, it was noted that the Malayan tiger origin country needs to design and organise strategic and management plans for the captive animal facilities, as indicated by 78.6% of the respondents agreeing (Figure 4).

### 3.2. Anthropogenic Disturbances

Within about seven decades of persecution and prime habitat loss [22,23], the population experienced a rapid decline from 3000 individuals in the 1950s down to less than 200 animals in 2019. The Malayan tiger population was estimated at 500 individuals in 1990 [24] and had declined to 250–340 individuals by 2013 [6]. Unfortunately, the drastic trend has continued to nosedive to a critical level of near population crash. The estimated wild Malayan tiger population in 2018 was fewer than 200 individuals [15] (Table 1). Thus, the population lost approximately 60% of the wild Malayan tiger population in the last three decades since 1990.

### 3.3. Human-Wildlife Conflict and Roadkill

Considering the human–tiger conflict context, there were 652 cases of conflict recorded during 2008–2019. The yearly trend reveals that the number of conflict cases is decreasing. The conflict cases fluctuated from 86 cases per year in 2008 to 41 cases per year in 2019 (Table 2).

Viewing the data recorded from 2008–2019, the Department of Wildlife and National Parks (DWNP) has captured four conflict tigers and lost one tiger due to accidental roadkill. Malaysia also estimated that the country has lost approximately 39 tigers to illegal poaching from 2008–2018 [29] (Table 2).

Between 2008–2019, there were 99,954 cases of human–wildlife conflicts recorded in Malaysia, 652 cases involved the Malayan tigers (Table 2).

### 3.4. Roadkills

Regarding Peninsular Malaysia, there were 3386 cases of roadkills in nine years (2011–2019). The State of Johor recorded the highest roadkill numbers with 702 cases and the lowest recorded was one case in the Federal Territory (Table 3).

The government of Malaysia provided RM2.5 million to purchase land for corridors, habitat, rehabilitation, signs, road repairs, monitoring, and equipment (Table 4). The total cost of constructing 20 bridges and viaducts is unknown, but the Sungai Yu was RM89.9 million, Grik-Jeli RM60 million, and Pahang-Terengganu RM100 million.

## 4. Discussion

Over the past eight decades, Malaysians witnessed the extinction of the Java rhinoceros (*Rhinoceros sondaicus*) in 1932, the Banteng (*Bos javanicus*) in the 1950s, and the Sumatran rhinoceros (*Dicerorhinus sumatrensis*) in 2020 in Peninsular Malaysia [7,33,34,35,36,37,38]. The Malayan tiger is now on the brink of a population crash due to the small and reproductively-isolated individuals in fragmented habitats. A concerted effort is needed to protect the habitats, improve the numbers of apparently doomed populations of the Malayan tiger, create integrated captive breeding programmes, and ensure survivability for the next generations. Based on these results and records, we articulated our concerns and proposed strategic actions for the sustainable conservation of Malayan tigers over the next few decades.

### 4.1. Total Protection of Tiger Habitats

The in situ conservation policy of tiger conservation is well developed. Malaysia’s National Tiger Conservation Action Plan (NTCAP) is among the earliest comprehensive tiger actions in the region. The protection of forest habitat and the protection of tiger-prey species are among the actions enlisted in the NTCAP. Malaysia recognised the importance of the protected areas in biodiversity conservation and protection. All tiger habitats may be considered protected under the Wildlife Conservation Act (WCA) 2010 as part of the in situ conservation efforts, subject to agreements with the tiger range States within the Federal Constitution’s determinations. To secure the tiger prey species, Malaysia has issued a hunting moratorium on two main tiger prey species—the Sambar deer (*Rusa unicolor*) and the barking deer (*Muntiacus muntjac*) until 2021 [39]. The authority may consider implementing and extending the moratorium on hunting licenses to collect other wildlife to control wildlife harvesting.

Regarding ex situ conservation, the study has noted 93.1% of the respondents agree that scientific knowledge needs to be integrated into policy and practices (Figure 1). However, the integration of scientific knowledge for conservation policy and practice is still low (15.4% of always integration) (Figure 2) for Malayan tigers. Thus, the conservation community must carefully drive the Malayan tiger research and integrate the findings into the tiger policy. Malaysia is to draft a wildlife conservation policy to act as a working framework for wildlife management. The drafted policy shall revisit and insert a new conservation policy related to the anthropogenic disturbances, human–wildlife conflict, roadkill, and zoonotic diseases, that can save lives, protect livelihoods, and safeguard the Malayan tiger.

The survey also indicates that Malaysia, as the origin country of the Malayan tiger, needs to formulate a strategic plan (52% agreed) (Figure 3) to be followed by the global captive tiger facilities worldwide (78.6% agreed) (Figure 4). Currently, the captive Malayan tiger facilities play their roles based on their own interests and strong points. The authority may produce an international strategic plan that includes both an in situ and ex situ action plan. All zoos may use this plan to support the wild Malayan tiger population. Malaysia acknowledges it is a challenge to guarantee the survival of all animals under the current conditions of the natural habitats. However, Malaysia will continue to explore ways, using ex situ measures, to conserve biodiversity amongst large breeding groups of animals in zoos and captive animal facilities not only for tigers but, also, their prey species.

The Malayan tiger is listed under the Convention of International in Endangered Species of Wild Fauna and Flora (CITES). The CITES is an international agreement between governments to provide a sustainable international trade platform [40]. Regarding the national legislation, Malaysia gazetted the Malayan tiger in the International Trade in Endangered Species Act 2008 [Act 686] on 14 February 2008 to smooth the implementation of the Convention of International Endangered Species of Wild Fauna and Flora. Act 686 provides a legal basis for the CITES implementation in the country [41] by regulating the trade related to import and export. Concerning Peninsular Malaysia, the Wildlife Conservation Act 2010 [WCA 2010] has given the highest protection status for the Malayan tiger. Permission from the Minister of Energy and Natural Resources is required to keep the species.

The gazettement of Act, Enactment, and Ordinance in Malaysia has provided protection and conservation for the tiger [42,43]. However, there are a few challenges, for example, the lack of institutional enforcement capacity, a lack of cooperation among law enforcement agencies, and a lack of political will [42]. Those legal instruments need to be updated regularly to integrate scientific knowledge into the conservation and protection of the Malayan tiger (Figure 2).

### 4.2. Anthropogenic Activities (Mitigation Measures in Human–Tiger Conflicts)

Although studies show that the eco-certified logging concession in Peninsular Malaysia can preserve some of the threatened mammal species [44], logging’s negative side effect is alarming. The published report indicates the deforestation and biodiversity loss in Southeast Asia is the highest in the world [45,46], and we are losing our quality wildlife habitats. The concern arises further as it is reported that poaching (including snare trapping) of large felids and other species is increasing in Malaysia [29,47]. The national wildlife conflicts record also indicates that conflicts are unstable, and it is on an escalating trend over the last eight years (2012–2019) (Table 2). However, the human–tiger conflict shows a decreasing trend from 86 cases in 2008 to 41 cases in 2019, compared to the 84 human–tiger conflict cases in 1991 and 211 cases in 2002 [48]. The human–tiger conflict occurs when tigers pose a threat to the local communities’ livelihoods and safety, which leads to the ill-treatment of the species [49]. The frequency of conflicts indicates that tigers are getting closer to human settlement or human activities that might arise due to a prey shortage in the forest [6]. The decreasing trend is an alarm bell as the Malayan tiger population drops significantly–the trend indicating that we are losing our precious Malayan tiger. The authority needs to step up the mitigation measures by reviewing the moratorium on hunting, including reviewing the issuing of hunting licenses to secure Malayan tiger prey species. However, hunting licenses are still necessary for the case of human–wild boar conflicts and for research purposes.

**Figure 5:**
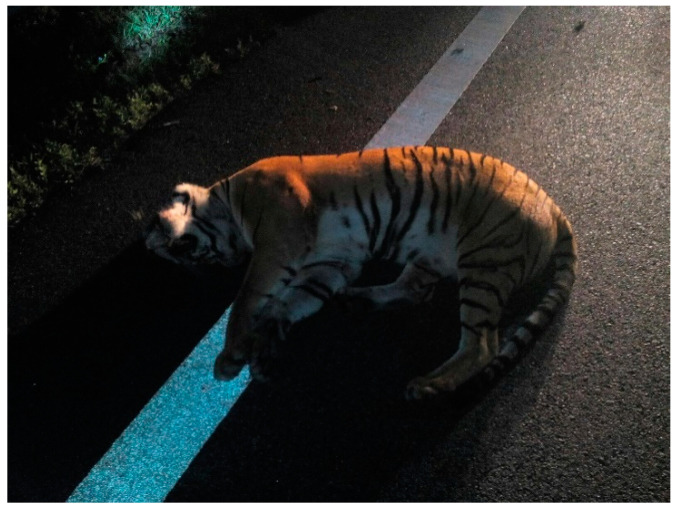
The female Malayan tiger was hit and killed by a high-speed vehicle along East Coast Highway 2 on 7 February 2016. Possibly due to the poor maintenance of highway fencing. (Photograph @Ahmad Ikhwan Zainuddin).

The animals will cross the road either for water, food, shelter, mates, or nesting sites, but road networks and fast-moving traffic have become dangerous. Roads and fast-moving traffic have killed a lot of wildlife, leading to local population losses [50,51,52,53]. The construction of road networks is the major contributor to the death of wildlife. Although there are 20 bridges and viaducts constructed [50] in Peninsular Malaysia, Peninsular Malaysia lost a Malayan tiger due to roadkill (Table 2) (Figure 5). Studies show that combining mitigation measures such as viaducts and fences has reduced roadkill [54]. Thus, the wildlife authority, highway authority, and road caretakers may need to step up and combine several mitigation measures to reduce roadkills.

During 2019, canine distemper disease infected the Malayan tiger, and the infectious disease may threaten the tiger population [55]. Malaysia has lost two wild Malayan tigers to the deadly canine distemper [8,9]. The first deadly canine distemper was reported on 19 July 2019 in a wild male Malayan tiger in Kampung Besul, Terengganu. The Department of Wildlife and National Parks (DWNP) managed to retrieve the individual tiger and provide various treatments but, sadly, the male tiger died on 23 July 2019 [8]. The second Malayan tiger was discovered dead at Ladang Aramijaya, Mersing Johor, on 1 May 2020, possibly due to canine distemper [9]. The canine distemper source cannot be confirmed but it is believed that the disease source is from other animals [8]. The newest deadly disease detected to infect the Malayan tiger is COVID-19. COVID-19 is a new pandemic disease caused by the SARS-CoV-2 virus (COVID-19 virus) [56]. COVID-19 was detected in the captive Malayan tiger on the 5 April 2020 in the Bronx Zoo, New York, United States of America [10,57,58,59]. The positive incidence of COVID-19 in the USA is very alarming for the local wildlife authority regarding the control of zoonotic pathogens jumping from humans to tigers in Malaysia [12]. The wild Malayan tiger population is at a crossroad, with confirmed canine distemper disease and potential COVID-19, for future survivability. The threats of canine distemper disease and COVID-19 can threaten Peninsular Malaysia’s seven cat species, including the Malayan tiger, in Malaysia’s natural habitat and zoos [11,12]. Both canine distemper and COVID-19 are critical issues to Malayan tiger conservation. The nature of transmission of COVID-19 from human to animal will significantly impact the Malayan tiger populations. Once the disease reaches the wild Malayan tiger in the forest, COVID-19 can spread among them [60,61]. Until the impact of COVID-19 on the Malayan tiger is available, Malaysia is suggested to review and follow the zoonotic disease preventive control measures strictly. Avoiding or minimising interaction between domestic animal reservoir hosts and endangered wildlife species is a strategy to curb the spreading of infectious diseases [62,63]. The villagers, as animal hunters, are the potential COVID-19 carriers. Thus, to manage COVID-19, the authority needs to control human–tiger conflict cases effectively. Tightened and effective mitigation measures are needed to control the spreading rate of canine distemper and COVID-19 to buy time until the emergence of COVID-19 vaccines [12].

### 4.3. Professional Staff and High Technology

The present research on tigers uses labour-intensive methods by counting and comparing tracks of vital activities (counting direct or indirect signs) [44,64] and using remote tracking or specialised equipment (camera traps) [44,65,66,67]. To improve the current methods, the country’s researchers may consider remote wireless biosensor network technology to detect volatile organic compounds released from the tiger urine and faeces. The current development of wireless biosensor technology can differentiate between humans and a large cat [68]. These large-scale networks (deploying small biosensor devices) claim to be able to gather information from the physical environment (temperature, sound, chemicals, seismic waves, infrared, still and motion video camera, or the presence of certain objects) and perform simple processing [68]. The remote system could provide early warning to the ground crew to provide real-time tracking to arrest poachers within the vicinity of the tiger habitats.

## 5. Constraints

We distributed the questionnaire before the emergence of the COVID-19 pandemic. The questions in the survey were mostly designed from the standard for animal ex situ management. We were aware that the questionnaire feedback was limited. There are 48 captive animal facilities globally, and ten facilities in Malaysia that own Malayan tigers. We attempted to reach out to all the facilities but only managed to gather responses from 14 facilities with 30 respondents. This study provides an early indication of ex situ Malayan tiger management as a basis for an expanded questionnaire study.

## 6. Conclusions

The habitat protection, sustainable financing, inclusive species policy, strategic plan, and action plan in situ and ex situ are the essential ingredients for successful Malayan tiger survival to avert possible extinction. Aside from managing the wild and captive populations, Malaysia needs to address the threat of infectious disease (canine distemper and COVID-19) to the Malayan tiger. Malaysia’s wildlife authority needs to move swiftly to protect and avoid the critically endangered Malayan tiger from being infected and falling into the COVID-19 chain. The Malayan tiger conservationists need to continue collaborating, supporting, and co-managing its ex situ management as part of assisting and increasing its wild Malayan tiger population. Malaysia needs to set an integrated strategy for sustainable conservation of all the wildlife, including the Malayan tiger as a keystone species. Additionally, the authority also could explore, modify, and adapt the Giant Panda conservation model’s sustainable financing mechanism. The Giant Panda conservation efforts to bring the species back from extinction risk have proved that its conservation model is successful and workable [69,70].

Malaysia is working toward the United Nations Sustainable Development Goals (SDGs), designed to end poverty, protect the planet, and ensure that all people enjoy peace and prosperity by 2030 and achieve the SDG 15. The SDG 15′s goal is to protect, restore, and promote sustainable use of terrestrial ecosystems, sustainably manage forests, halt and reverse land degradation, and halt biodiversity loss. Future research should identify potential sites for reintroduction of Malayan tigers in protected areas. Alternatively, intensive management of the wild population could be established in an electrified fenced area. These areas could ensure the biosecurity of the genetic pool could be closely monitored while effectively controlling the biosafety against emerging infectious diseases or pandemic.

## Figures and Tables

**Table 1:** Estimated wild Malayan Tiger population in Malaysia.

Year	Estimate Population/Individuals	Source
1950s	3000	Locke [25]
1982	250	Khan, et al. [26]
1986	650	Khan [27]
1990	500	Topani [24]
1991	400	Abidin, et al. [28]
2013	250–340	Kawanishi [6]
2018	200	Abdul Halim, Mustapha and Ibrahim [16]

**Table 2:** Human–Tiger Conflicts in Malaysia 2008–2019.

Year	Human-Wildlife Conflict (Case)	Human-Tiger Conflict (Cases) ^1^	Captured due to Conflicts (Individuals)	Infected by Canine Distemper (Individuals)	Roadkill (Individuals) ^1^	Estimate Poached (Individuals) ^4^
2008	13,652	86	NA	NA	0	4
2009	13,244	82	NA	NA	0	5
2010	9281	87	0.2.0 ^2^	NA	0	8
2011	8029	62	NA	NA	0	6
2012	5602	65	NA	NA	0	5
2013	5628	69	0.1.0 ^3^	NA	0	1
2014	6456	38	NA	NA	0	0
2015	6236	29	NA	NA	0	1
2016	6741	27	1.0.0 ^2^	NA	1	4
2017	7428	35	NA	NA	0	2
2018	7902	31	NA	NA	0	3
2019	9755	41	NA	1	0	NA
2020	NA	NA	NA	1	0	NA
Total	99,954	652	4	2	1	39

^1^ Department of Wildlife and National Parks (DWNP) [30], ^2^ Abdul Halim, Mustapha and Ibrahim [16], ^3^ DWNP [31], ^4^ Wong and Krishnasamy [29], NA (Not available).

**Table 3:** Wildlife roadkills in Malaysia from 2011 to 2019.

States	2011	2012	2013	2014	2015	2016	2017	2018	2019	Total
Perak	100	58	60	83	91	57	48	27	33	557
Johor	16	78	99	113	90	113	79	65	49	702
Kedah	NA	NA	126	192	98	62	58	43	30	609
Kelantan	27	8	1	6	6	30	19	13	8	118
Terengganu	1	43	35	26	101	104	23	36	43	412
Pahang	20	15	2	11	2	18	29	124	159	380
Selangor	3	63	25	9	1	NA	2	2	2	107
N. Sembilan	NA	NA	9	24	77	50	45	47	78	330
Perlis	6	NA	23	20	14	16	4	NA	1	84
Melaka	NA	NA	10	15	6	20	5	8	5	69
P.Pinang	NA	NA	NA	3	5	5	3	NA	1	17
F. Territory	NA	1	NA	NA	NA	NA	NA	NA	NA	1
Total	173	266	390	502	491	475	315	365	409	3386

NA (not available).

**Table 4:** Estimated Total Development Cost.

Parameter	MYR (Million)	Percentage %
Primary linkages	1545.4	61.6
Secondary linkages	963.2	38.4
Total	2508.6	100.0

Data were adapted from the Department of Town and Country Planning (DTCP) [32].

## Data Availability

Data are reported in the Results and Supplementary Materials sections of this paper. Upon request, additional information can be obtained from the corresponding authors.

## Supplementary Materials

The following are available online at https://www.mdpi.com/article/10.3390/ani11041032/s1, Supplementary File 1: The detailed information about the questionnaire. Upon request, information can be obtained from the corresponding authors, Supplementary File 2: The analysed data for the questionnaire survey.
