# Supplementary material for: Panthera tigris jacksoni Population Crash and Impending Extinction due to Environmental Perturbation and Human-Wildlife Conflict
Source: Animals (Basel). 2021 Apr 6;11(4):1032. doi: 10.3390/ani11041032 (PMC8067357; doi:10.3390/ani11041032)
Supplement: Supplementary file 1 [file animals-11-01032-s001.zip › supplementary/Appendix B_Item Analyzsed_Malayan Tiger Mgmt Strategy_Conflict_AnimalsFormat_13022021.docx]

**Appendix 2**

**The analysed data for the questionnaire survey**

| Item No. | Items | N | | Mean | Median | Std. Deviation | Minimum | Maximum |
| --- | --- | --- | --- | --- | --- | --- | --- | --- |
|  |  | Valid | Missing |  |  |  |  |  |
| C7.39 | The animal host country needs to provide conservation policy or strategy or species management plan to the animal captive institution. Figure 4 | 28 | 2 | 4.25 | 4.50 | 0.887 | 2 | 5 |
| C7.40 | The scientific knowledge of species *ex-situ* management has well integrated into the species conservation policy and practice. Figure 1 | 29 | 1 | 4.52 | 5.00 | 0.634 | 3 | 5 |
| C8.16 | The integration of species captive management scientific knowledge into the species conservation policy and practice. Figure 2 | 26 | 4 | 3.46 | 4.00 | 1.104 | 1 | 5 |
| C9.19 | The species strategic, management plan and procedures research implemented by your institution. Figure 3 | 25 | 5 | 3.48 | 4.00 | 0.963 | 1 | 5 |

The analysed data for the questionnaire survey

| Item No. | Policy items | Percent | | | | | |
| --- | --- | --- | --- | --- | --- | --- | --- |
|  |  | Very unimportant | Unimportant | Neither unimportant nor important | Important | Very important | Total |
| C7.39 | The animal host country needs to provide conservation policy or strategy or species management plan to the animal captive institution. Figure 4 | 0 | 3.6 | 17.9 | 28.6 | 50.0 | 100.0 |
| C7.40 | The scientific knowledge of species ex-situ management has well integrated into the species conservation policy and practice. Figure 1 | 0 | 0.0 | 6.9 | 34.5 | 58.6 | 100.0 |
|  |  | Never | Rarely | Occasionally | Often | Always | Total |
| C8.16 | The integration of species captive management scientific knowledge into the species conservation policy and practice. Figure 2 | 7.7 | 7.7 | 30.8 | 38.5 | 15.4 | 100.0 |
|  |  | Strongly disagree | Disagree | Neither agree or disagree | Agree | Strongly agree | Total |
| C9.19 | The species strategic, management plan and procedures research implemented by your institution. Figure 3 | 4.0 | 8.0 | 36.0 | 40.0 | 12.0 | 100.0 |
