# Supplementary figures and images for: Panthera tigris jacksoni Population Crash and Impending Extinction due to Environmental Perturbation and Human-Wildlife Conflict
Source: Animals (Basel). 2021 Apr 6;11(4):1032. doi: 10.3390/ani11041032 (PMC8067357; doi:10.3390/ani11041032)

**Appendix 1 Questionnaire**

| **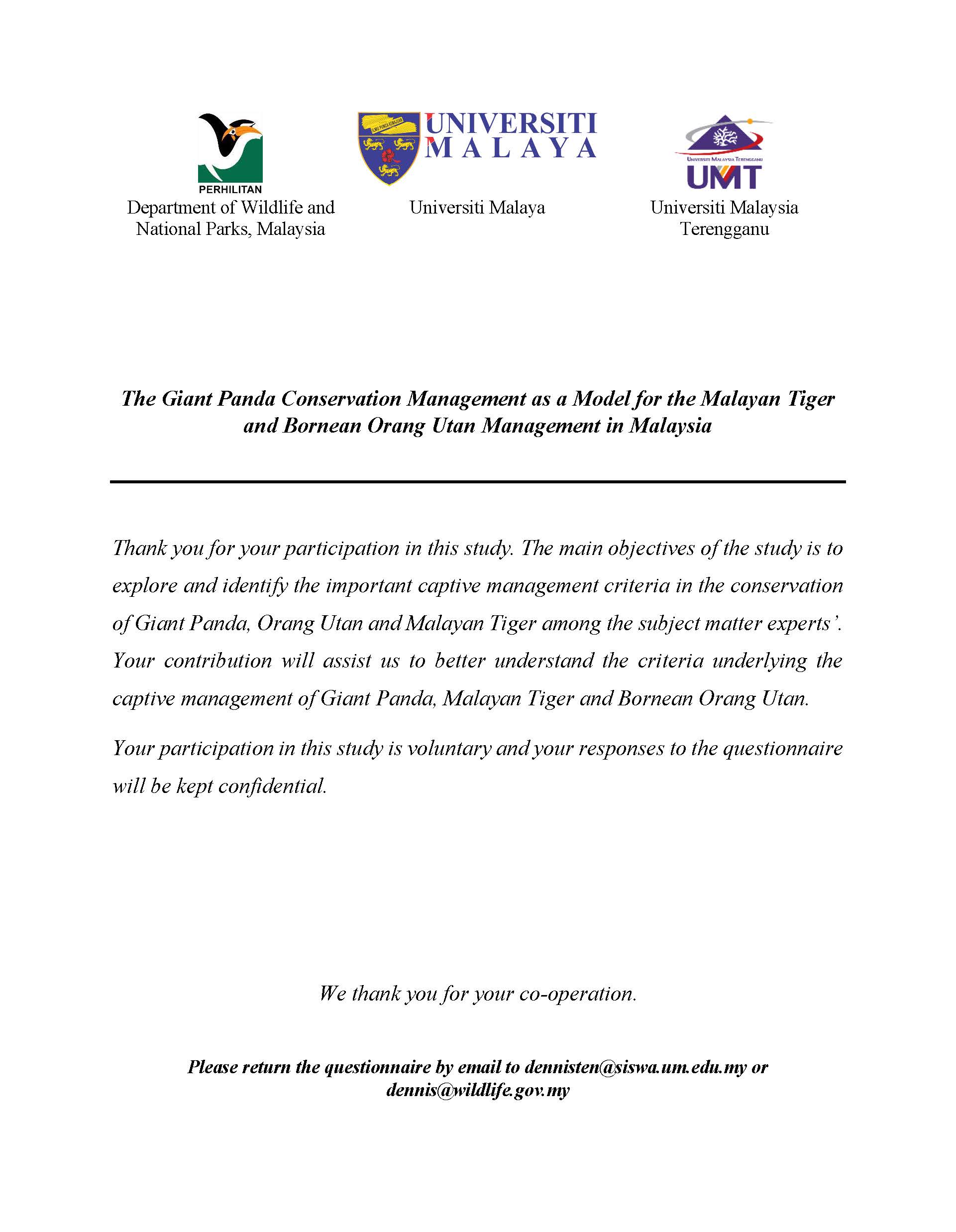** |
| --- |
| **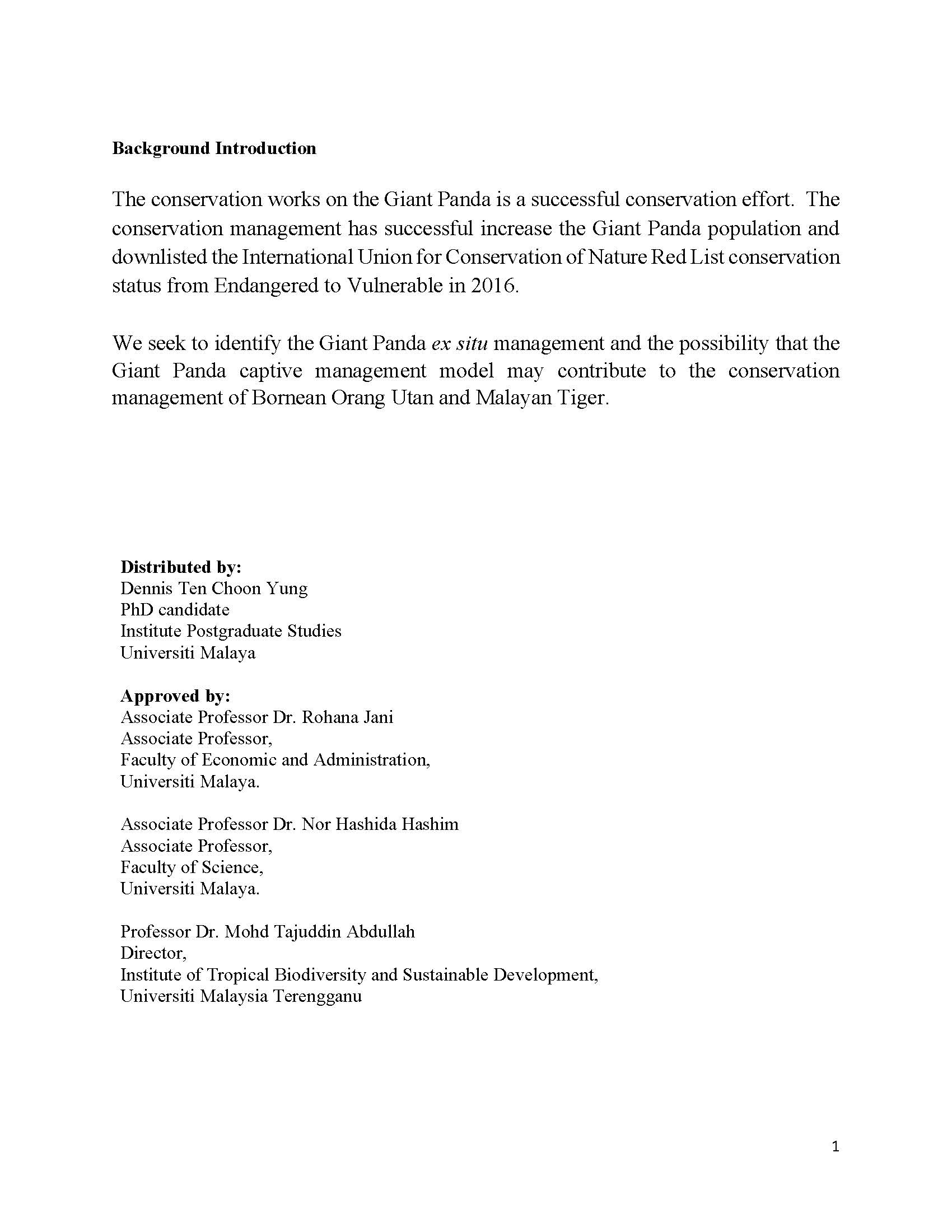** |
| **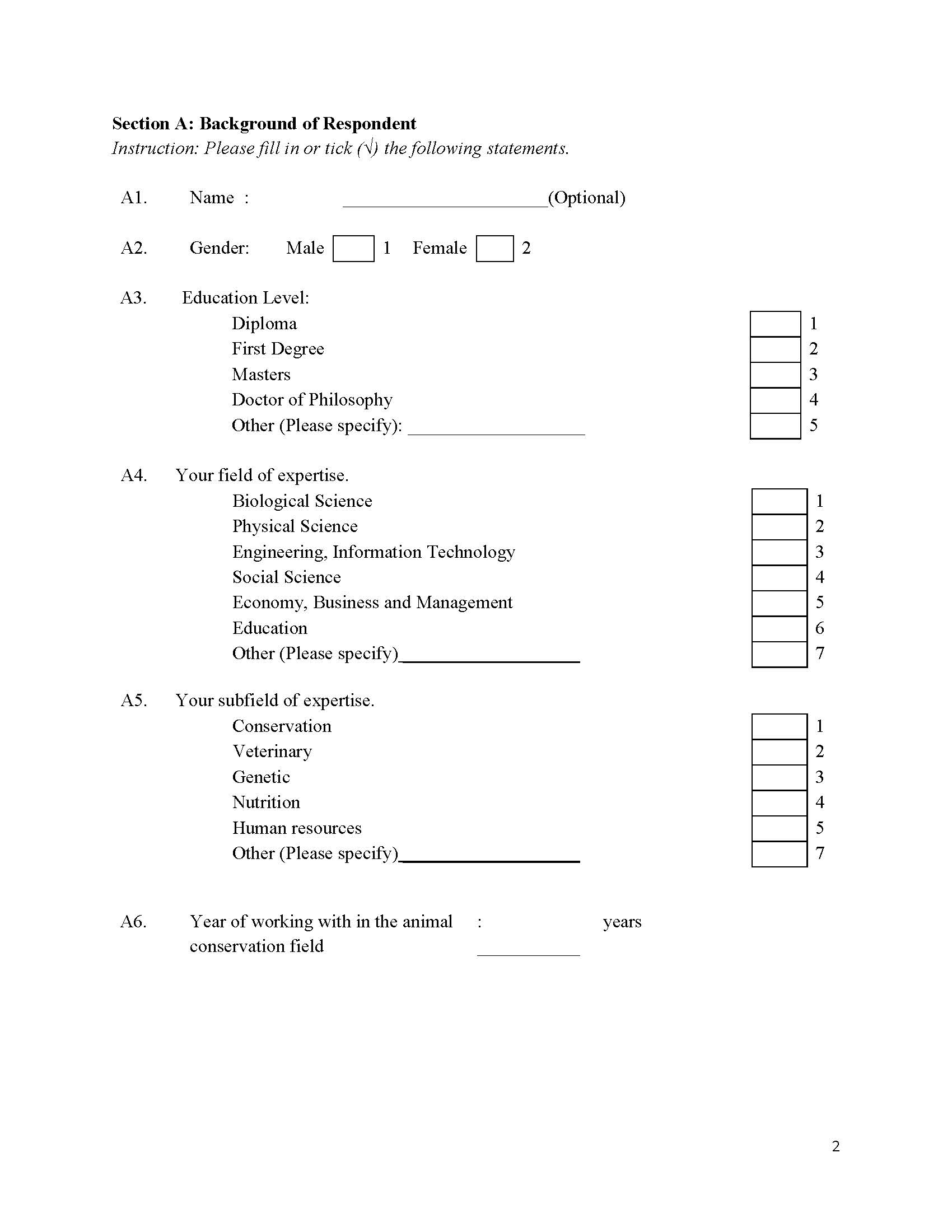** |
| **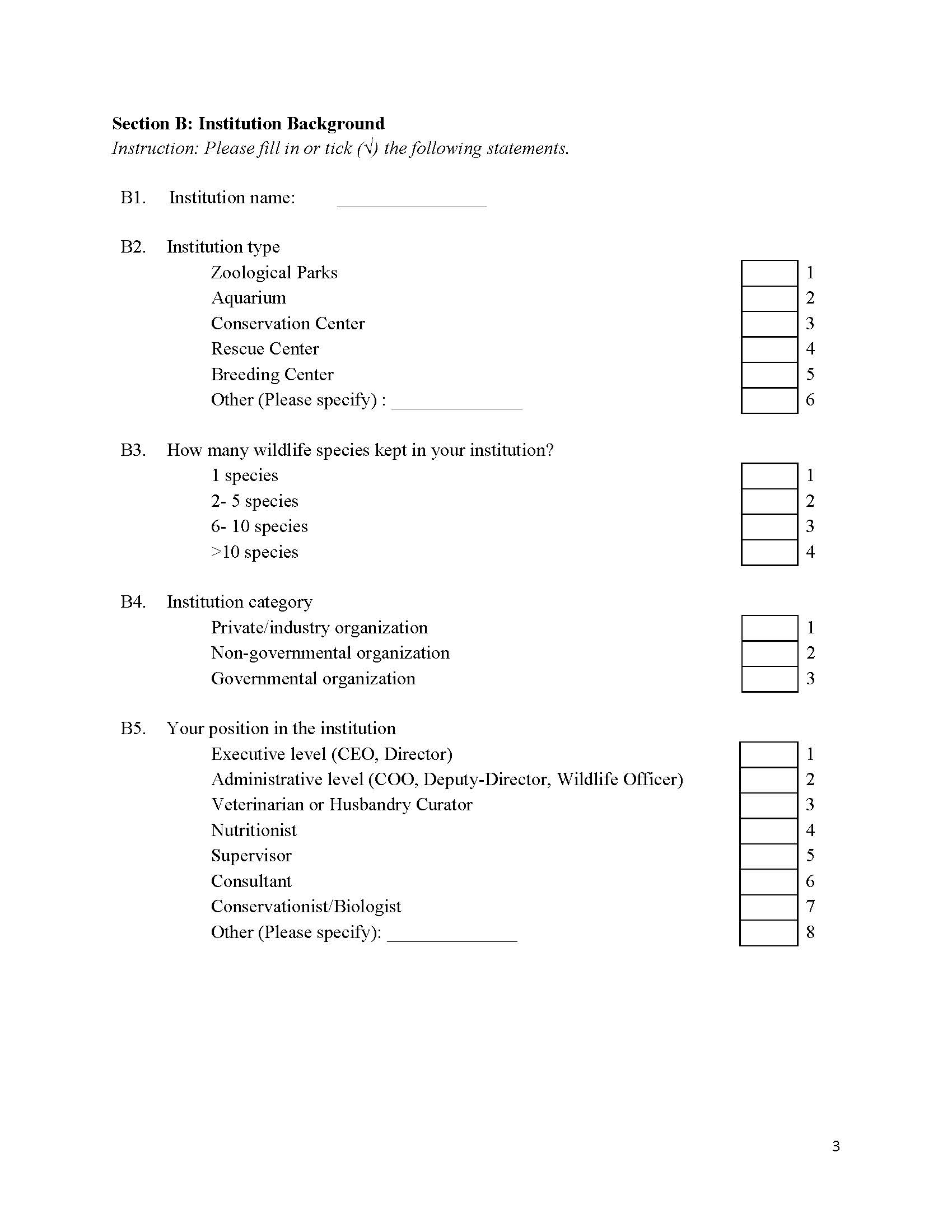** |
| **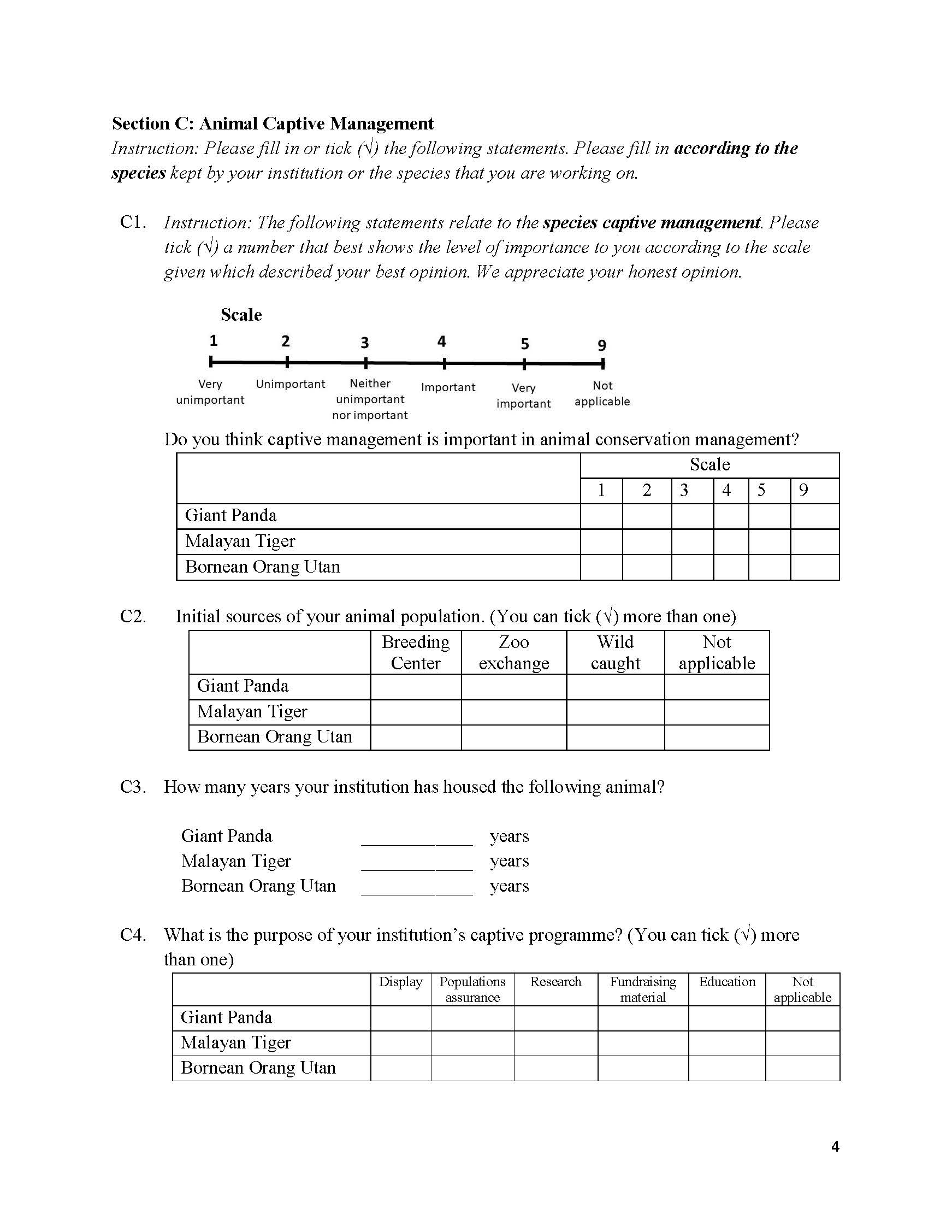** |
| **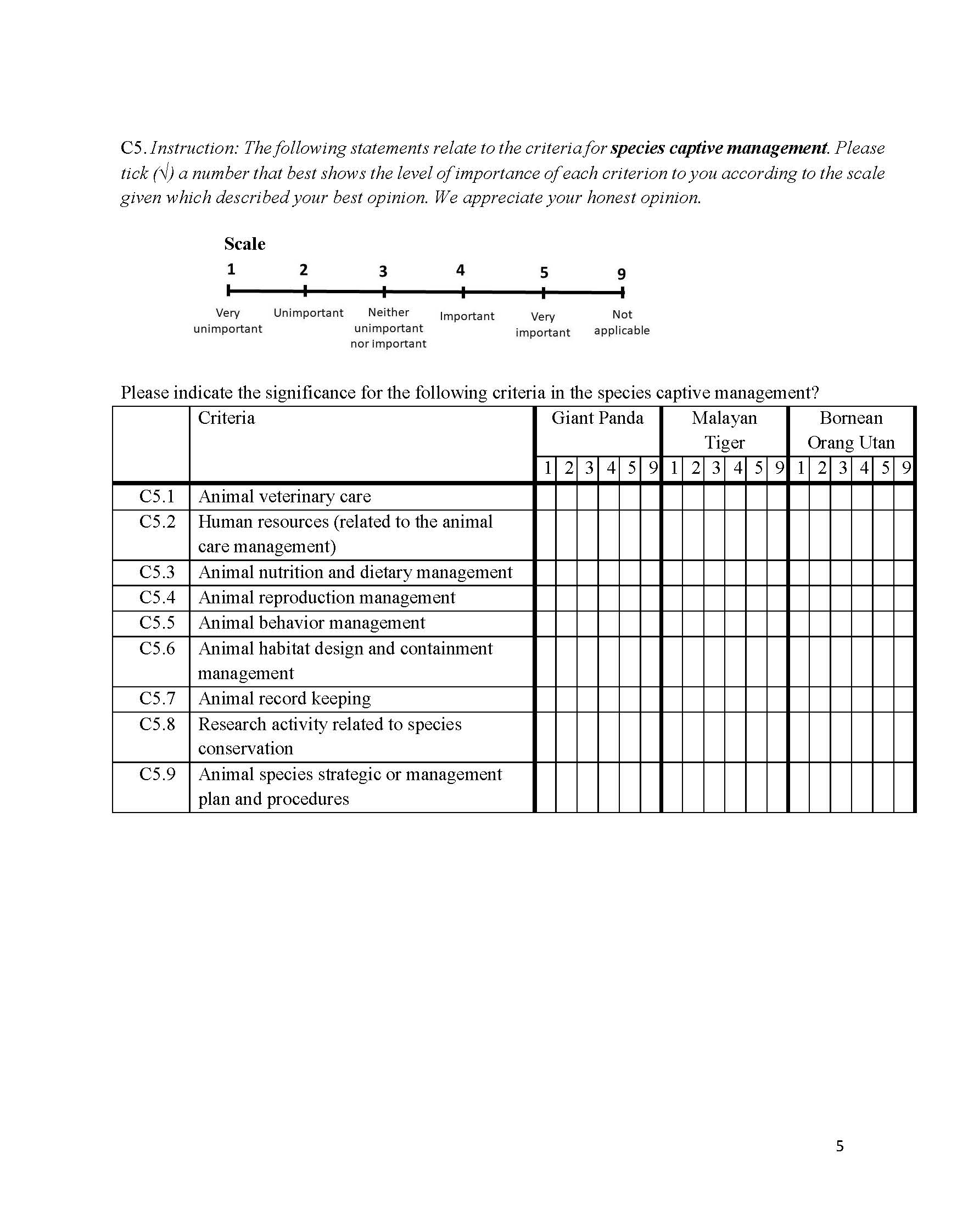** |
| **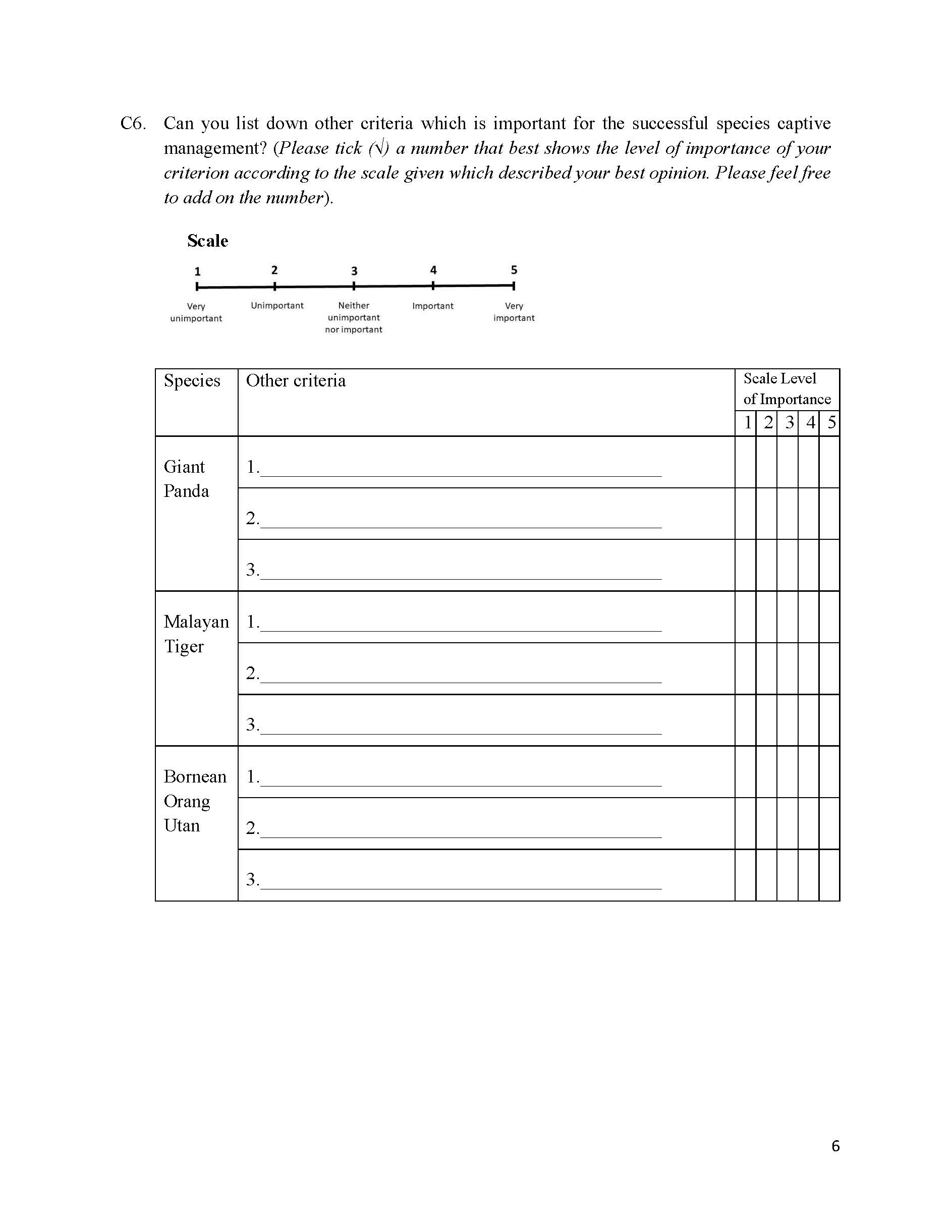** |
| **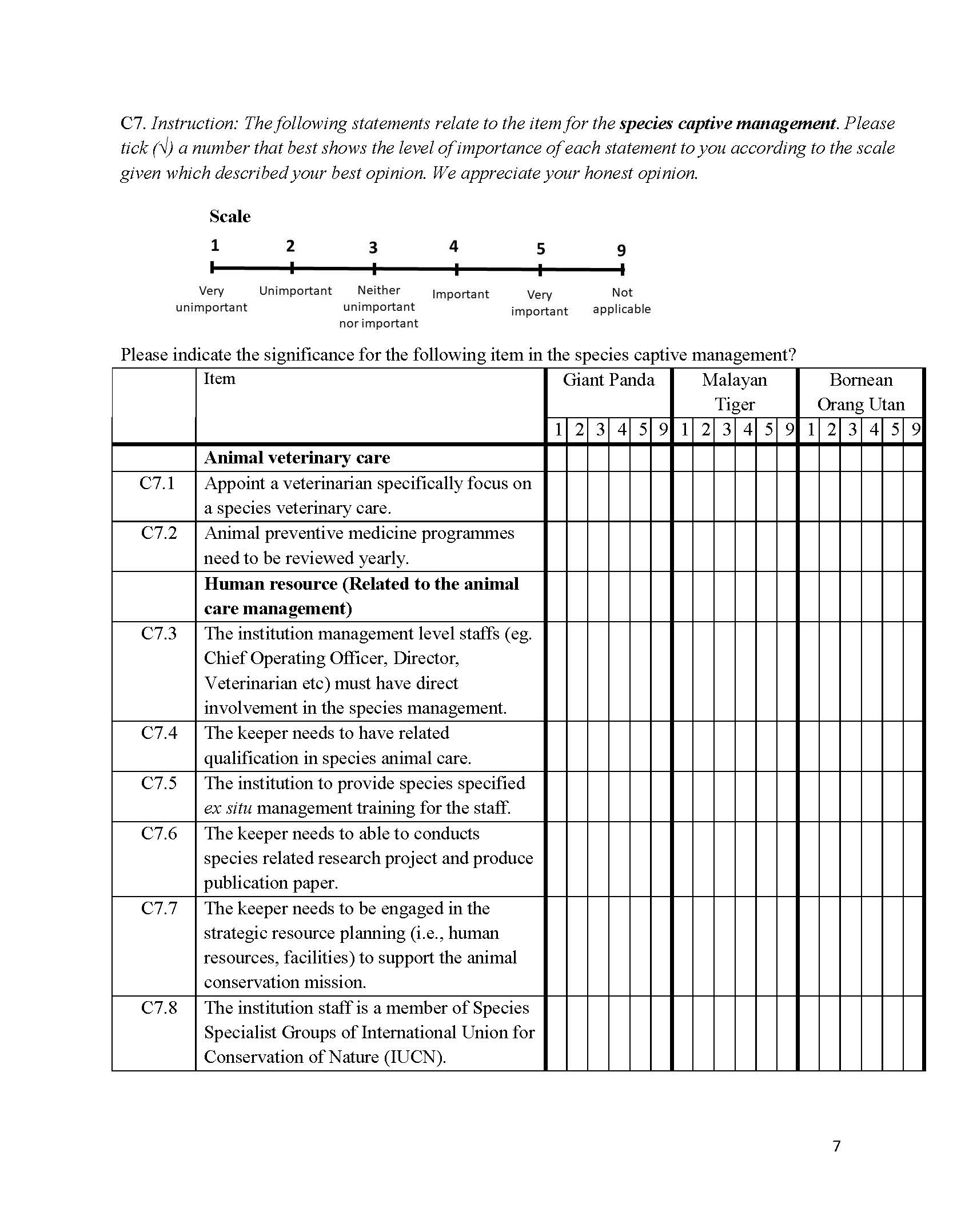** |
| **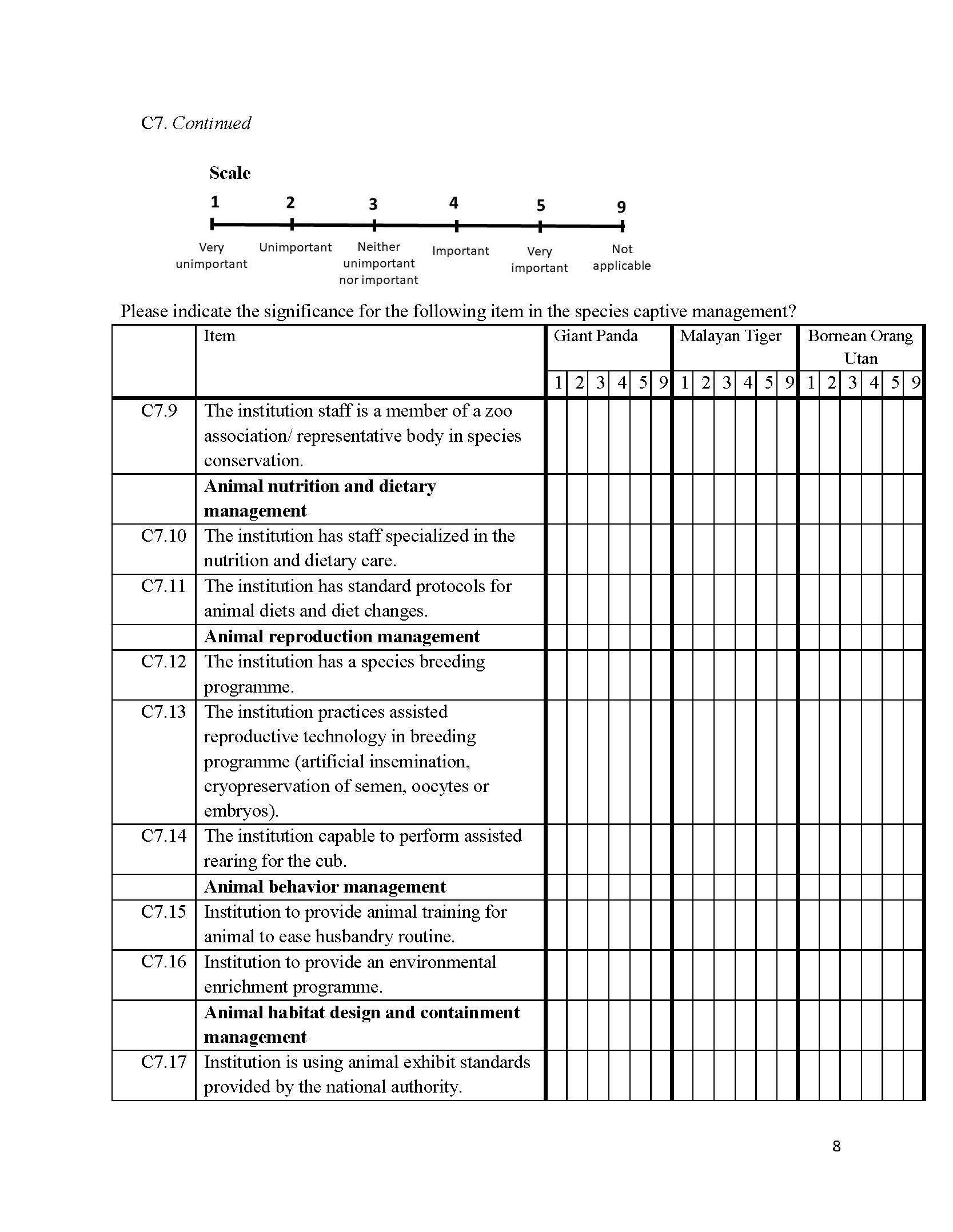** |
| **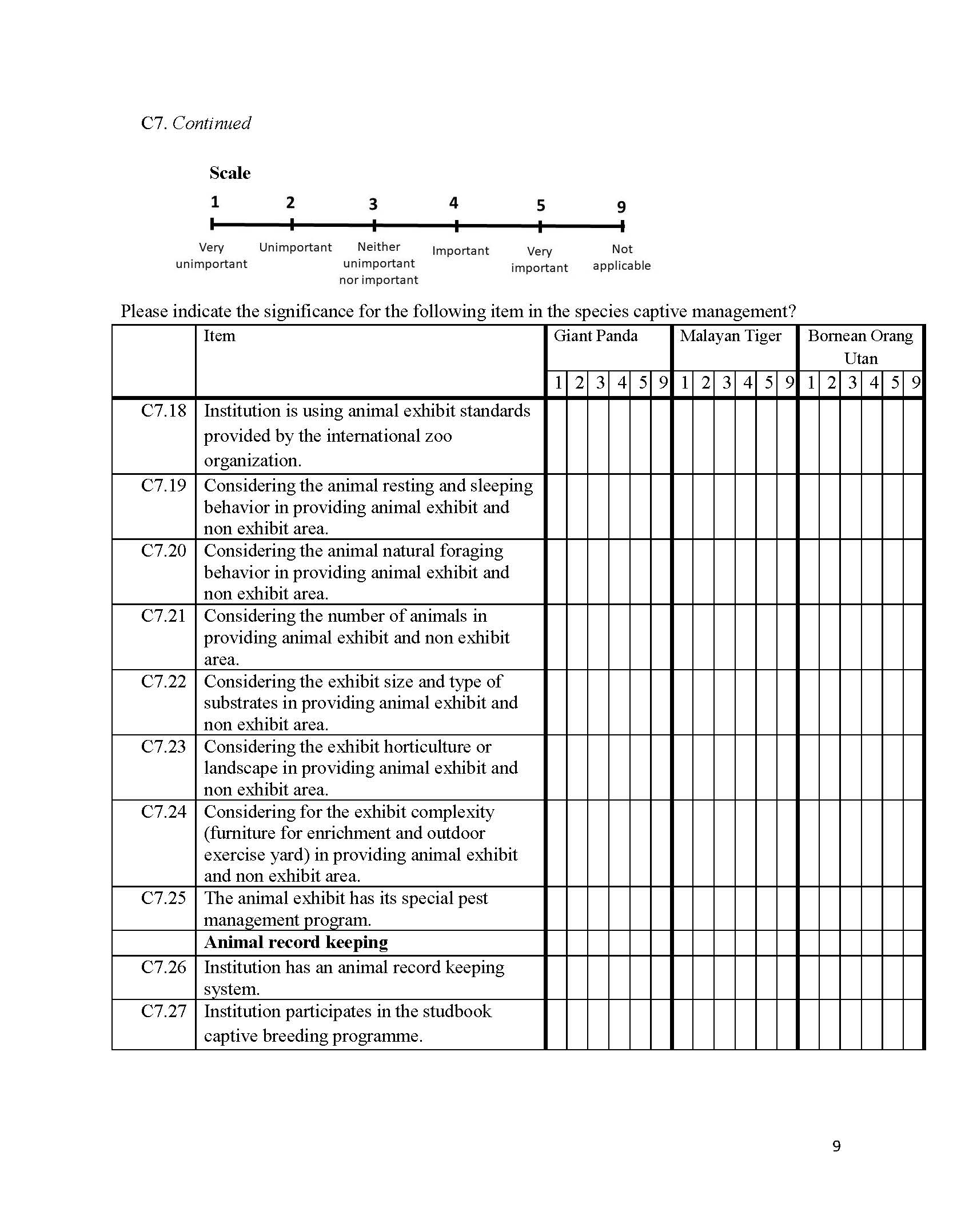** |
| **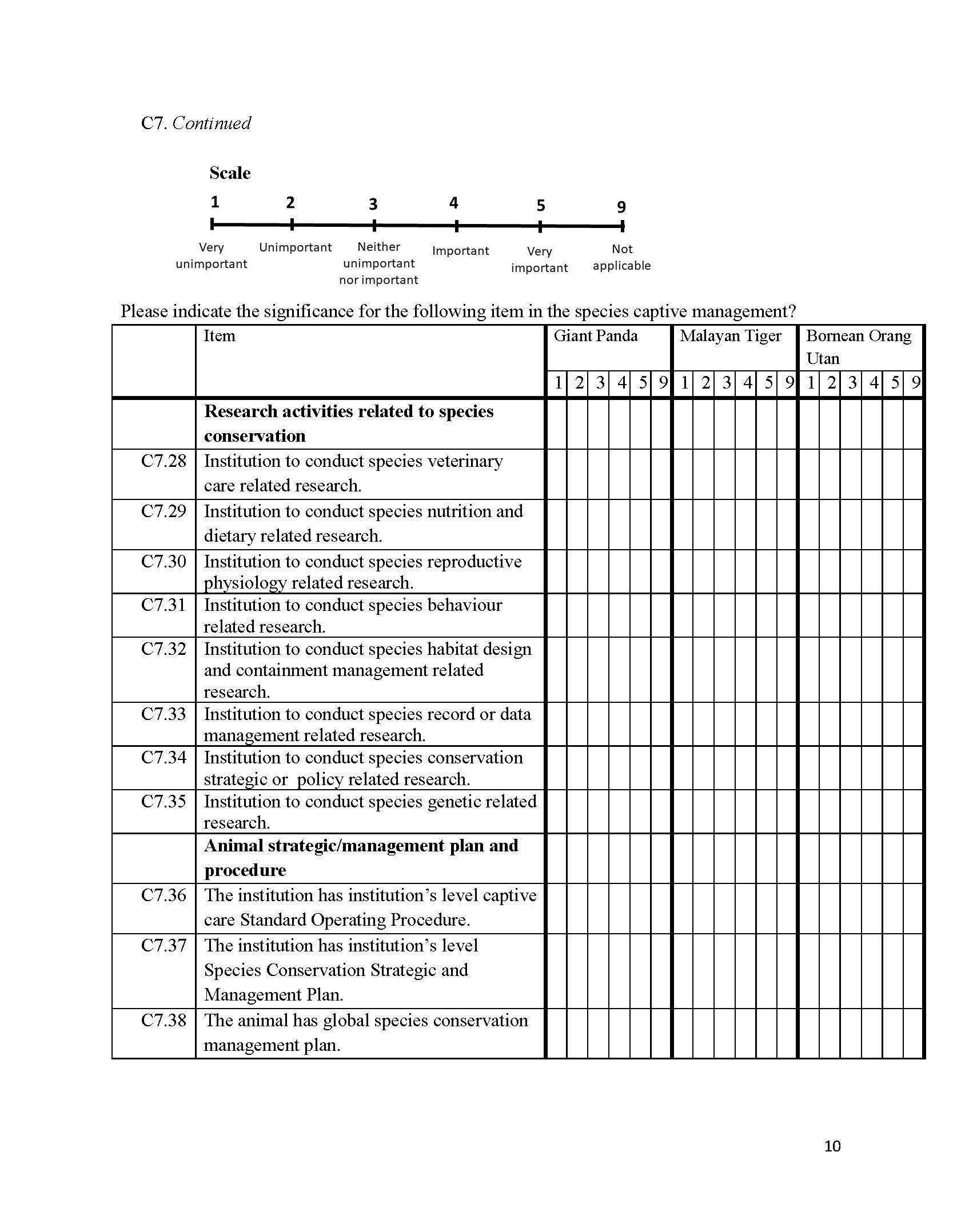** |
| **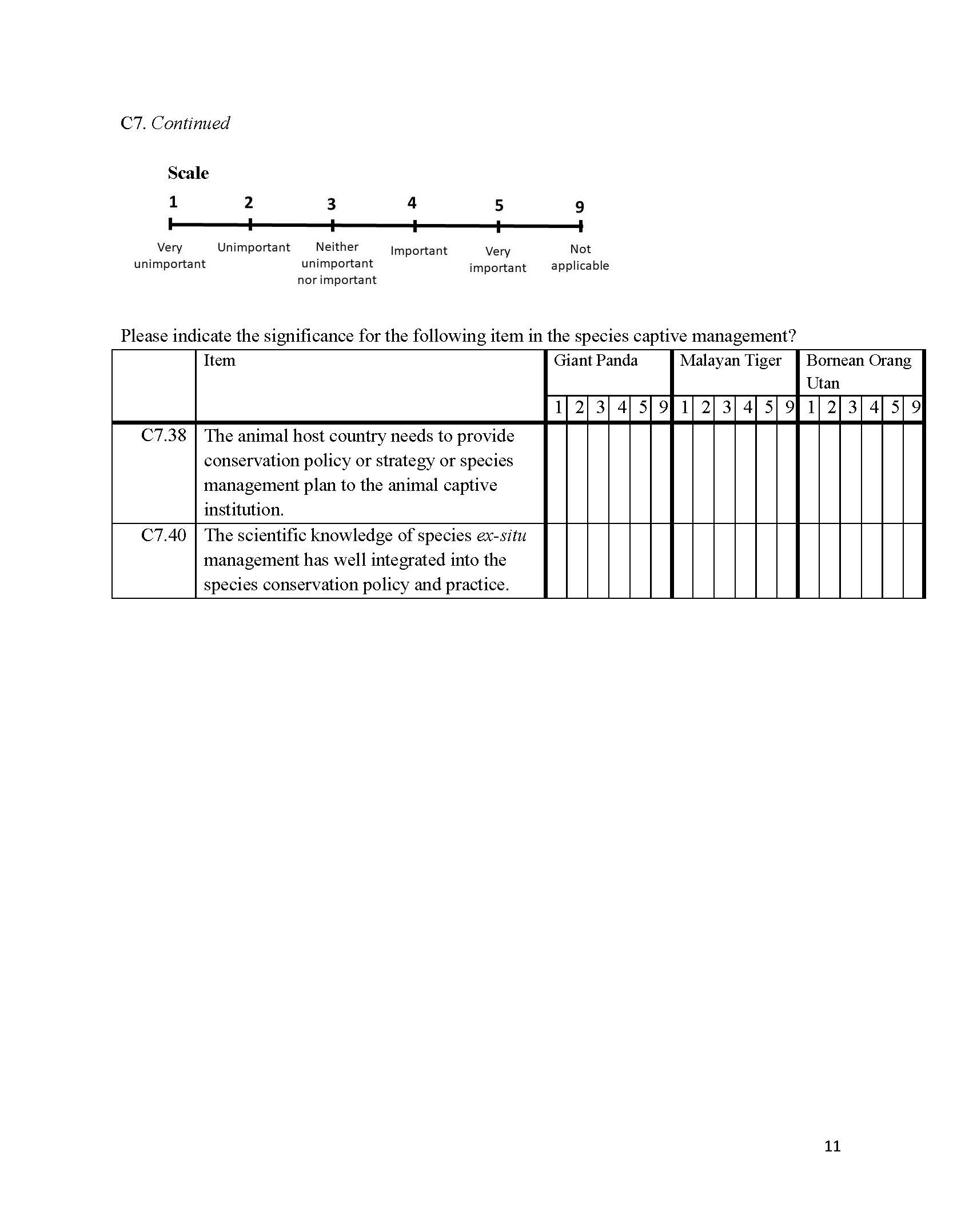** |
| **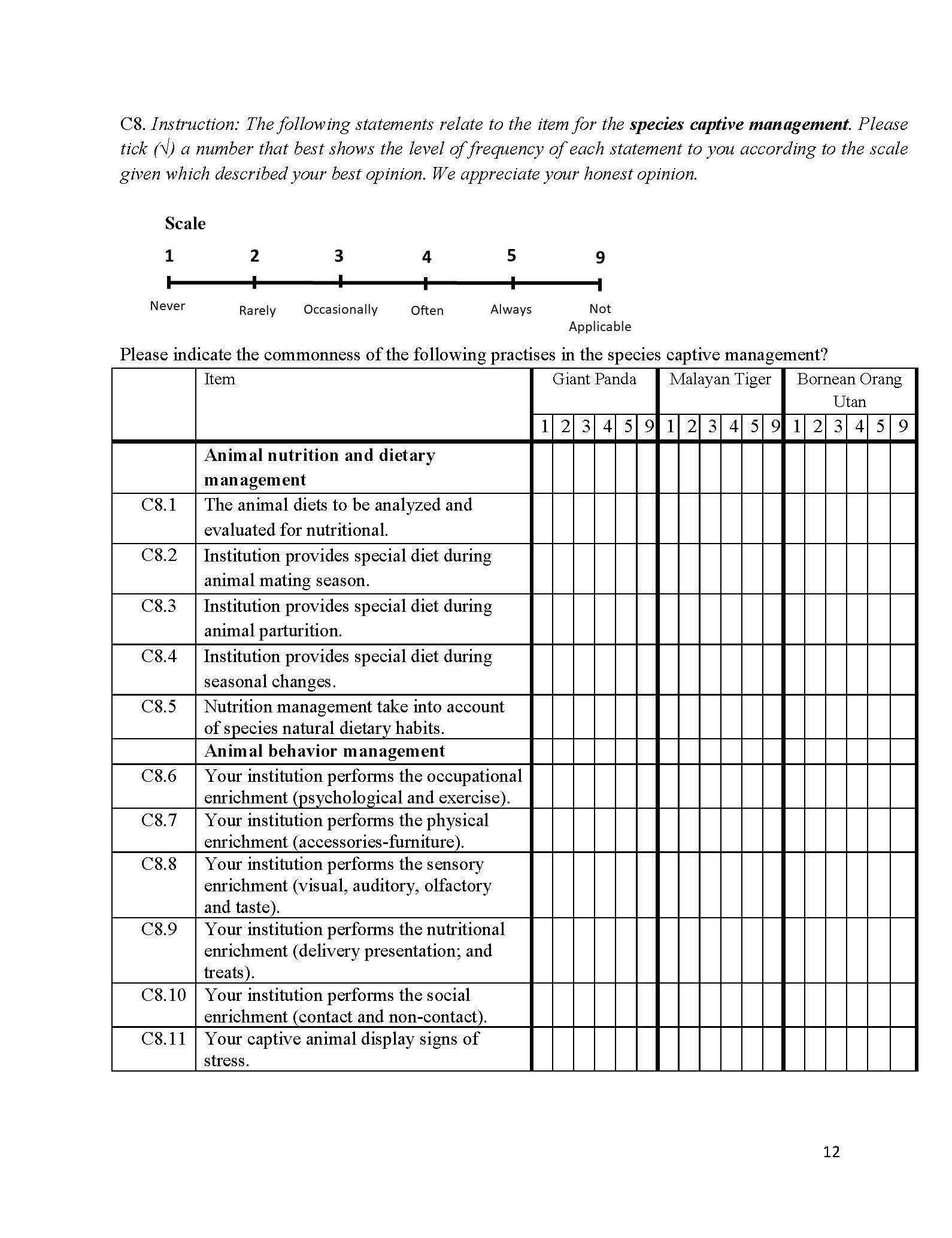** |
| **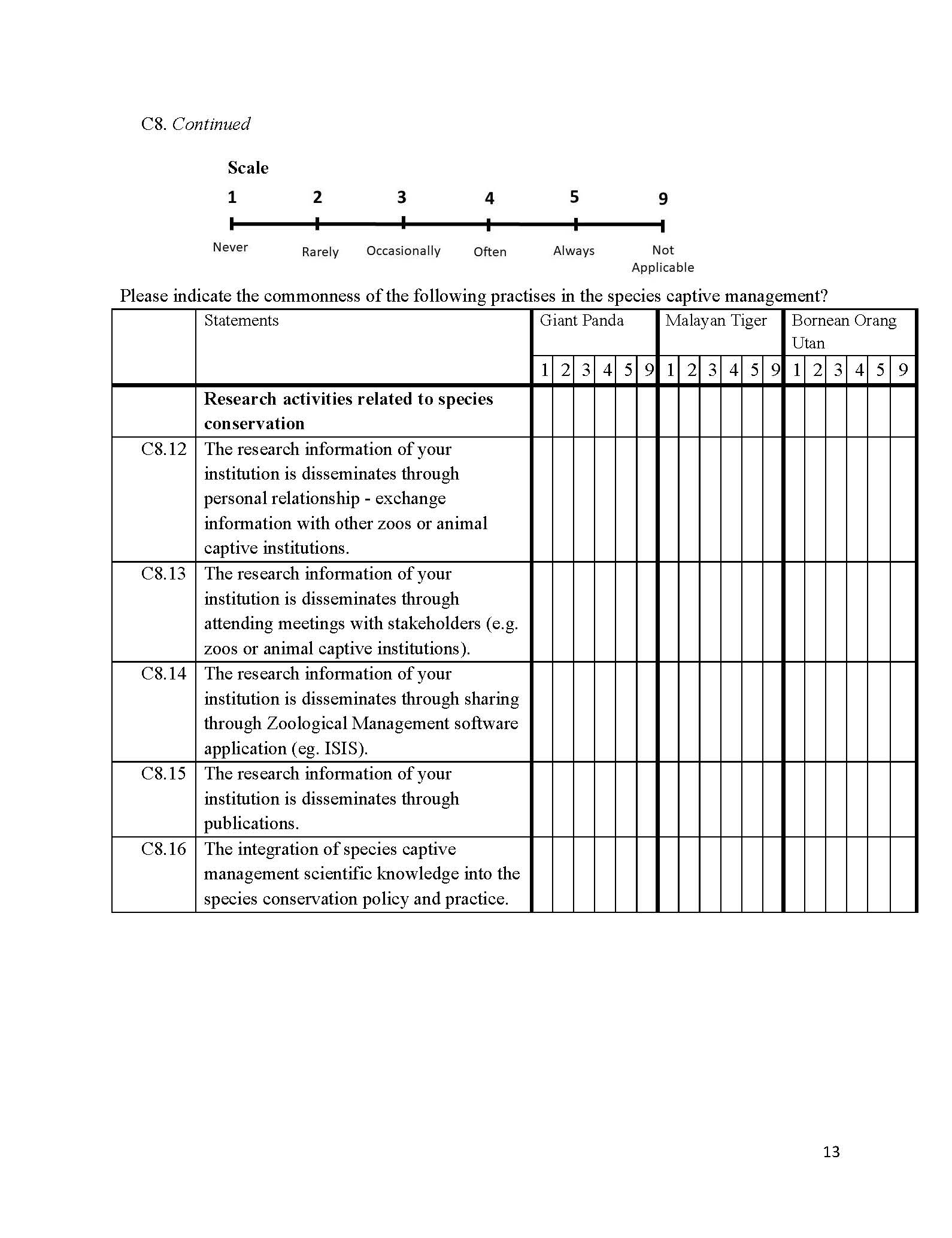** |
| **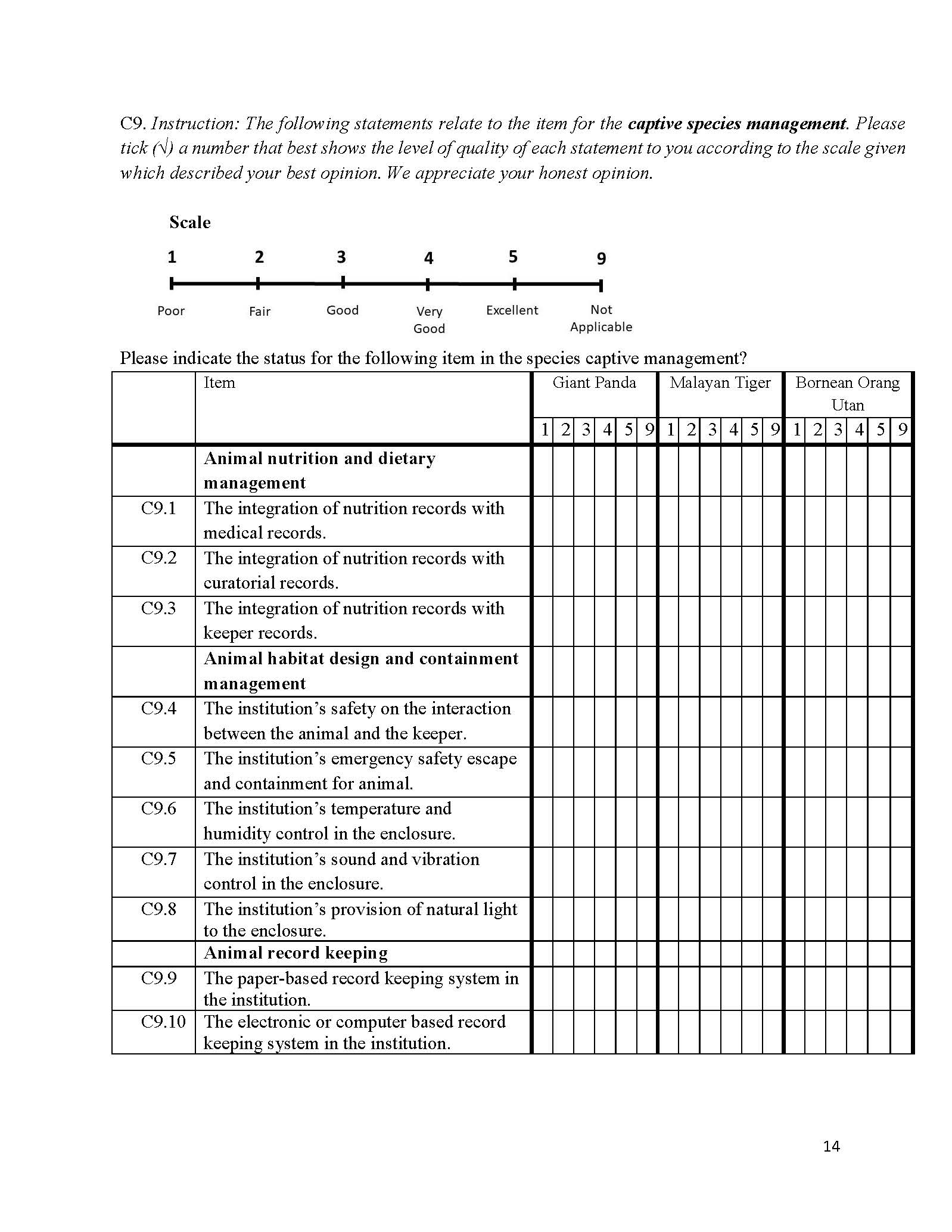** |
| **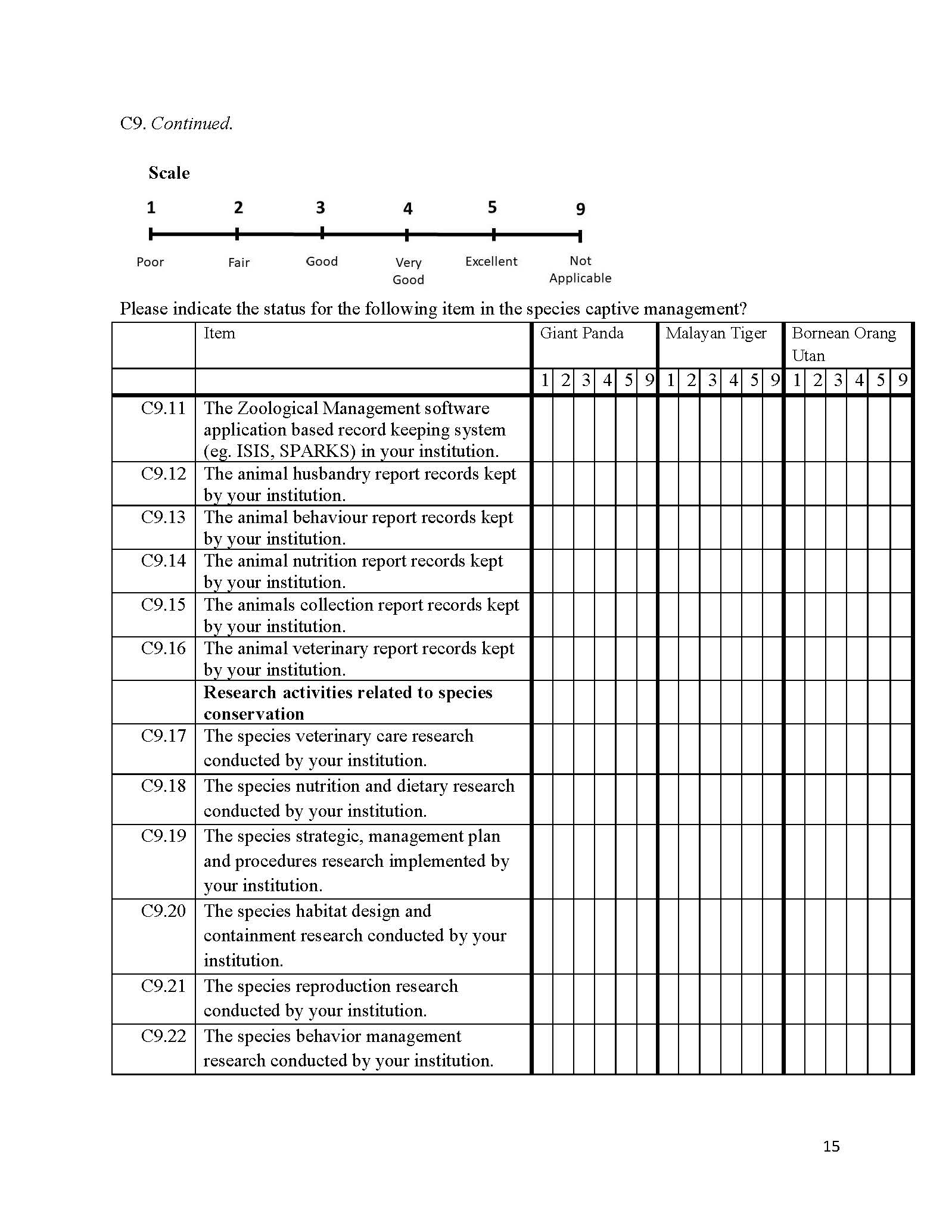** |
| **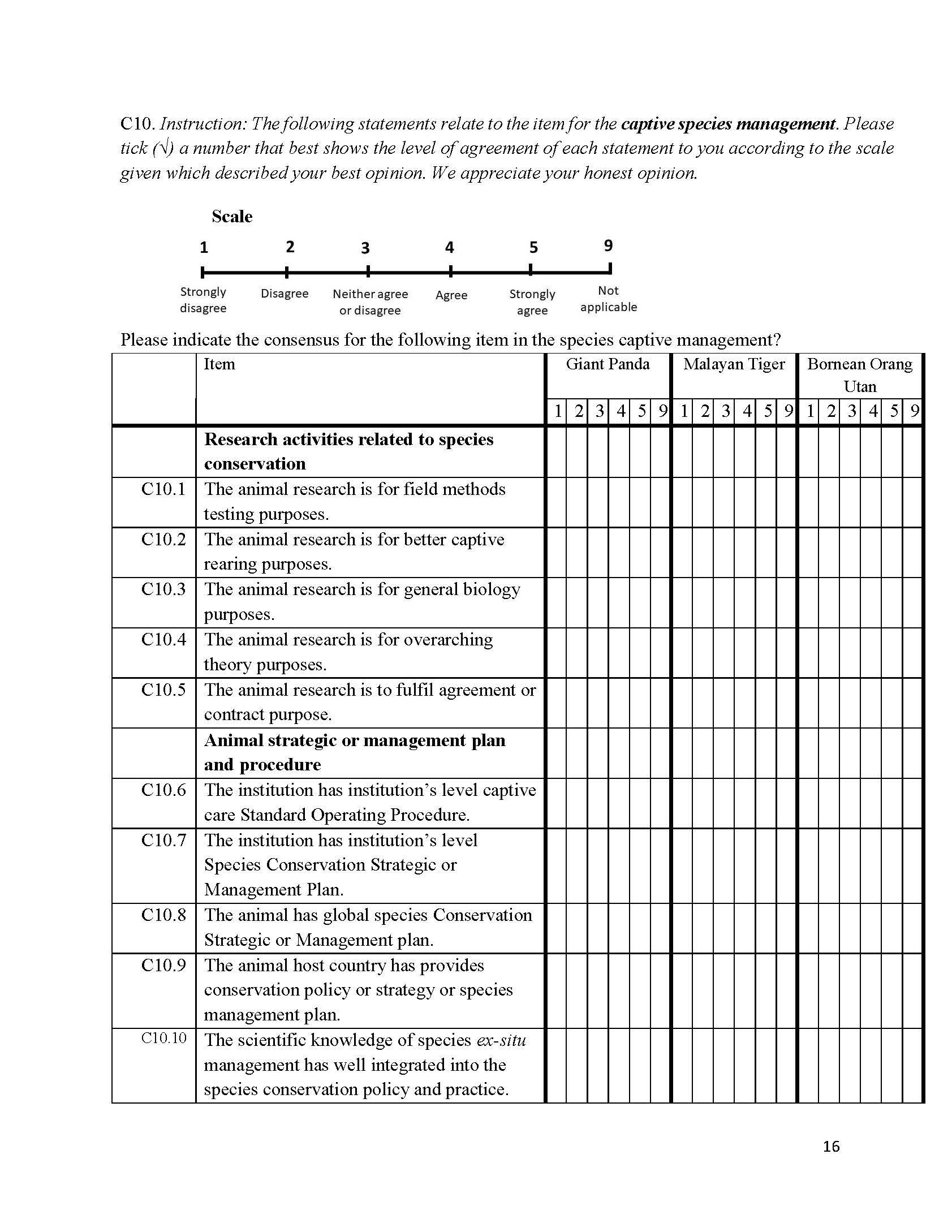** |
| **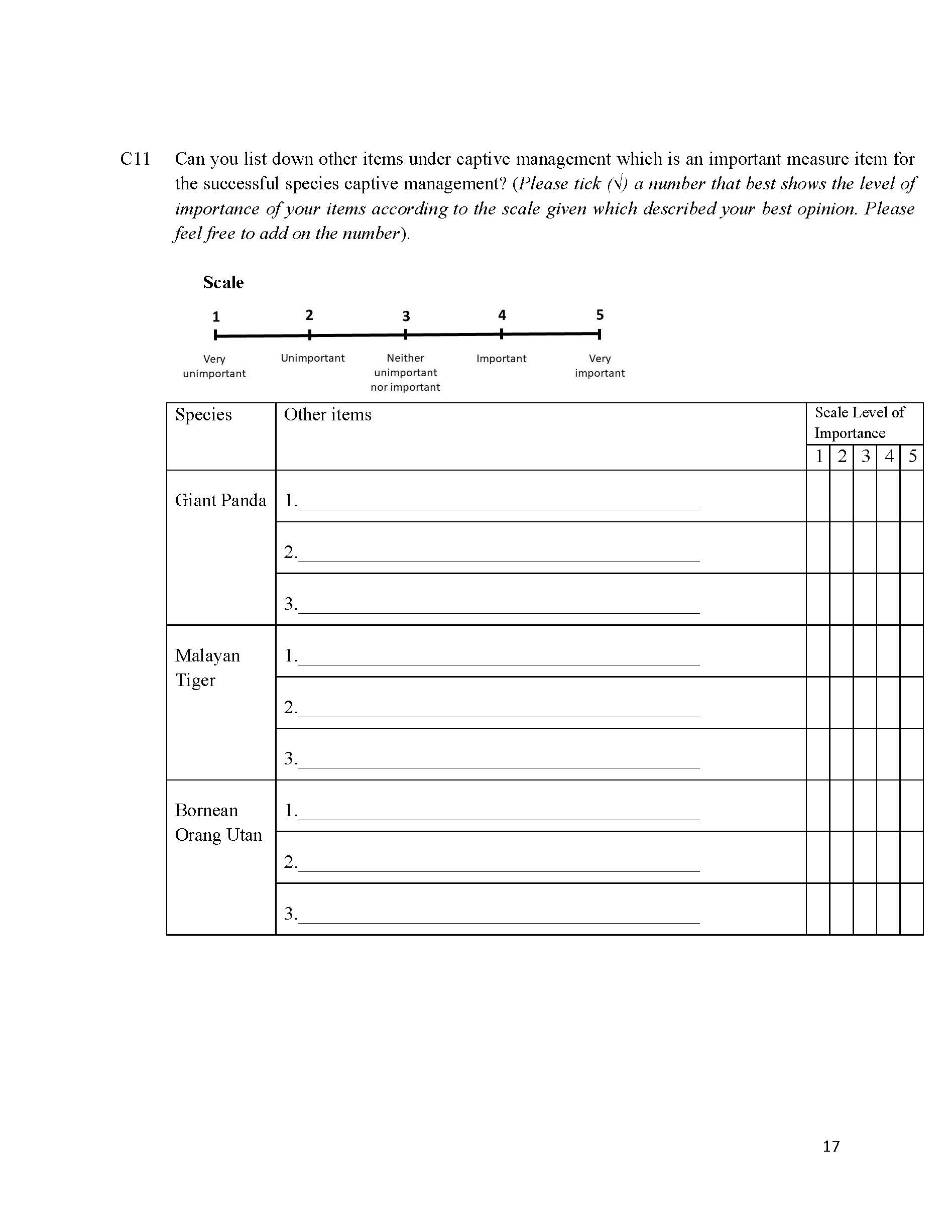** |
| **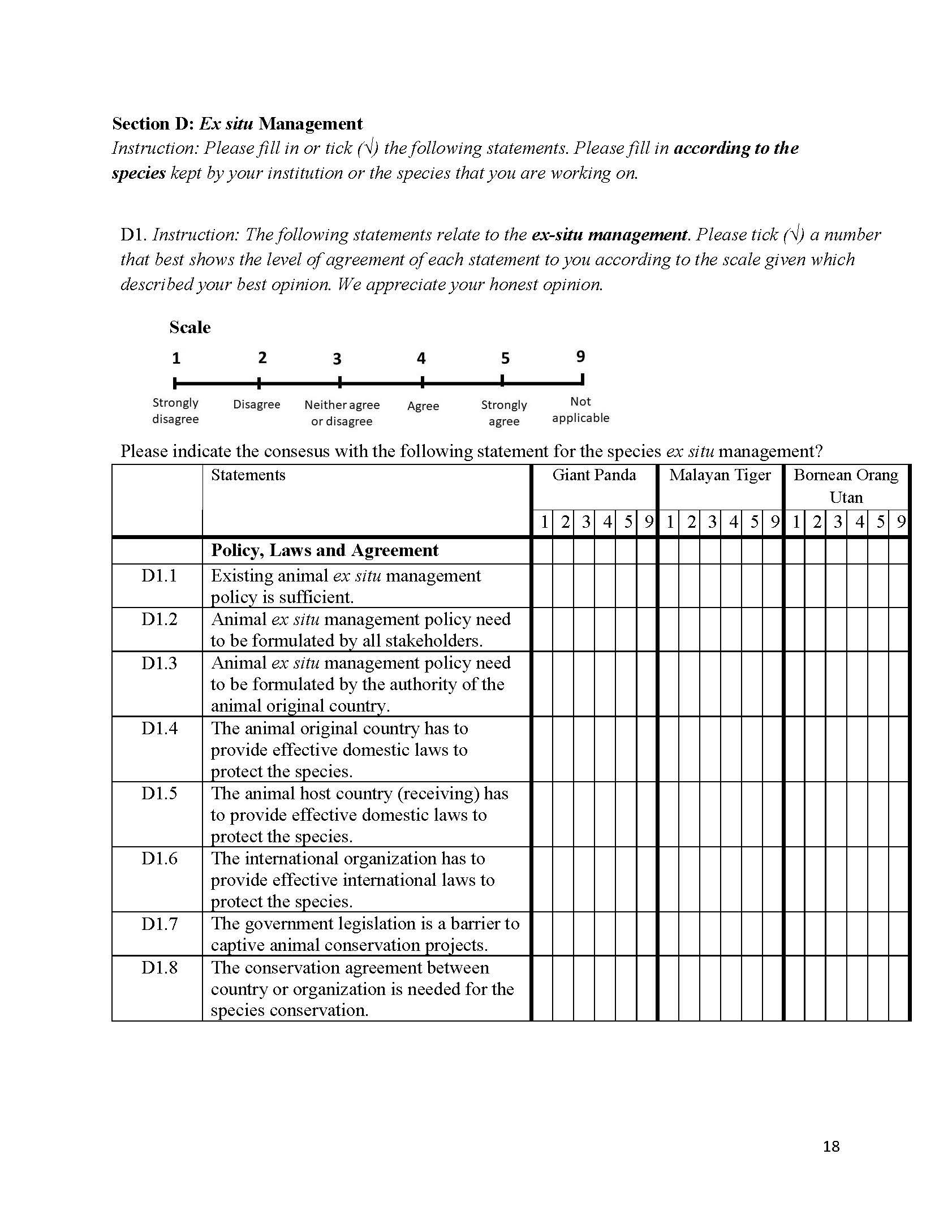** |
| **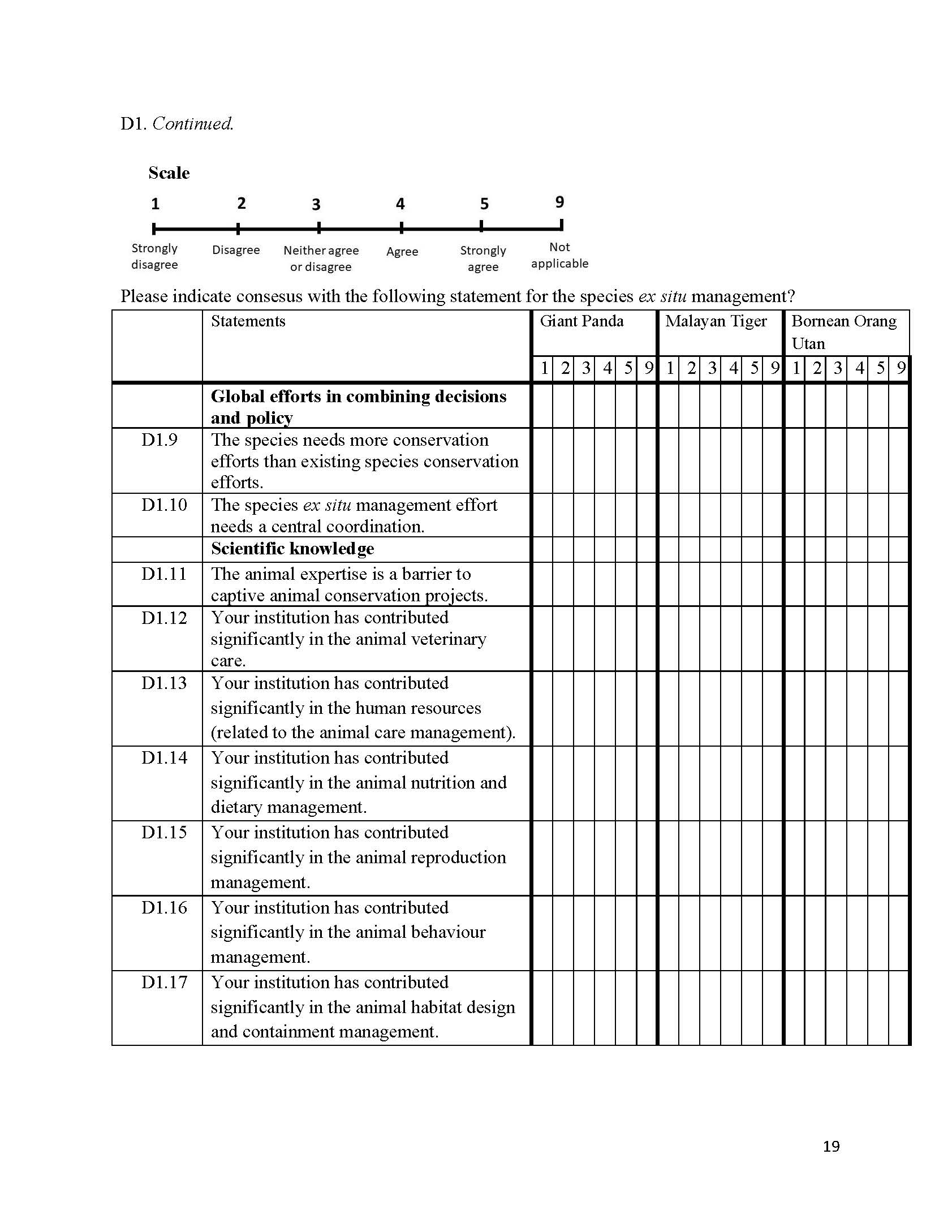** |
| **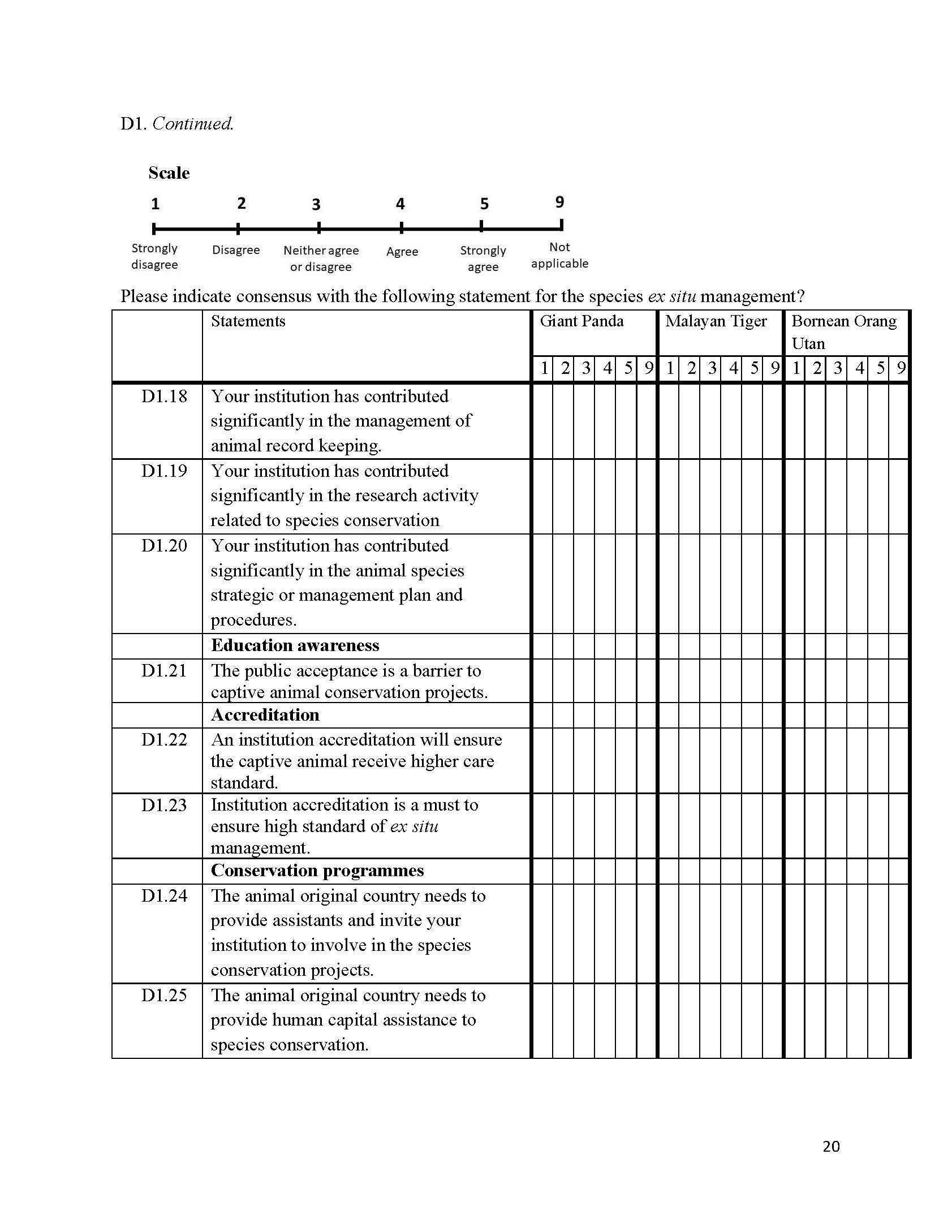** |
| **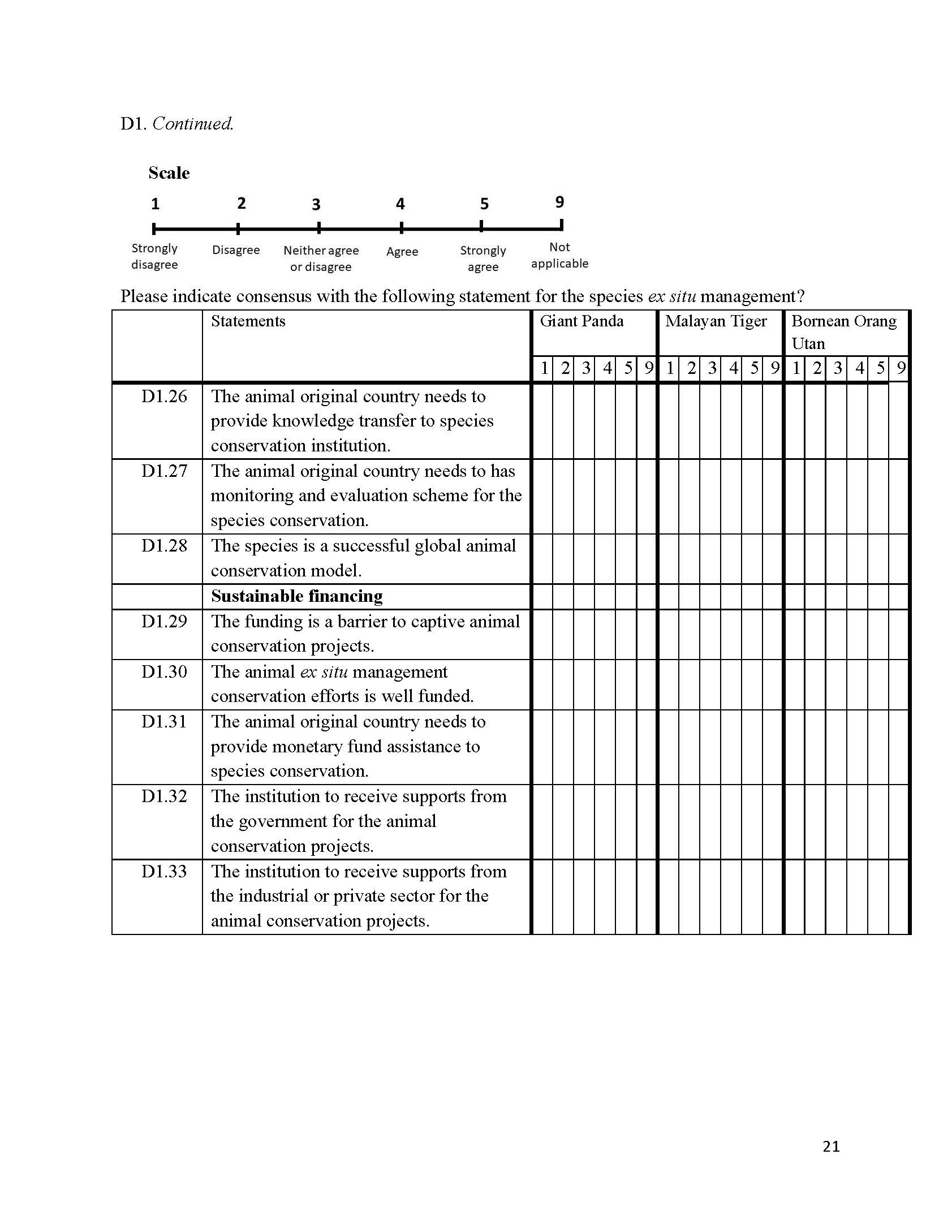** |
| **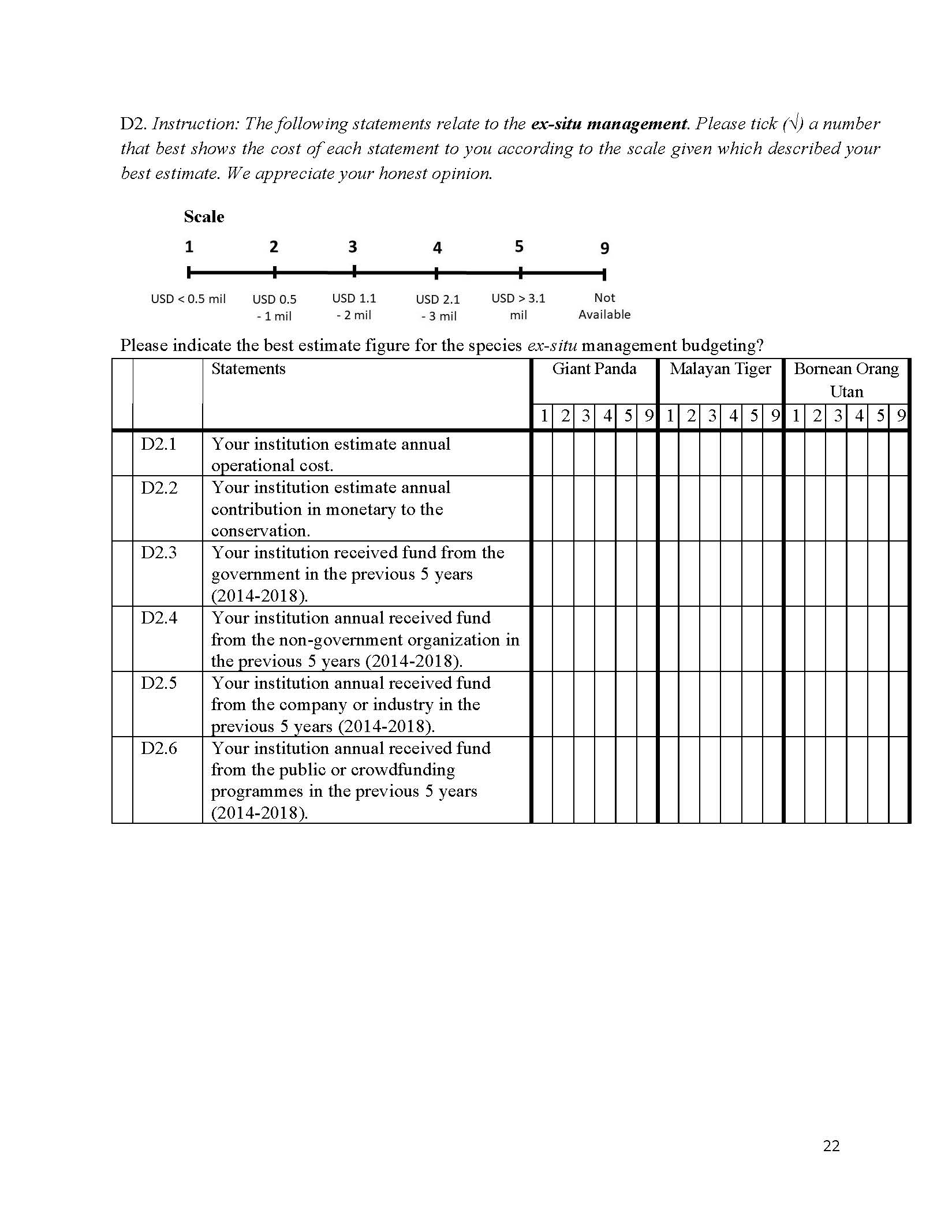** |
| **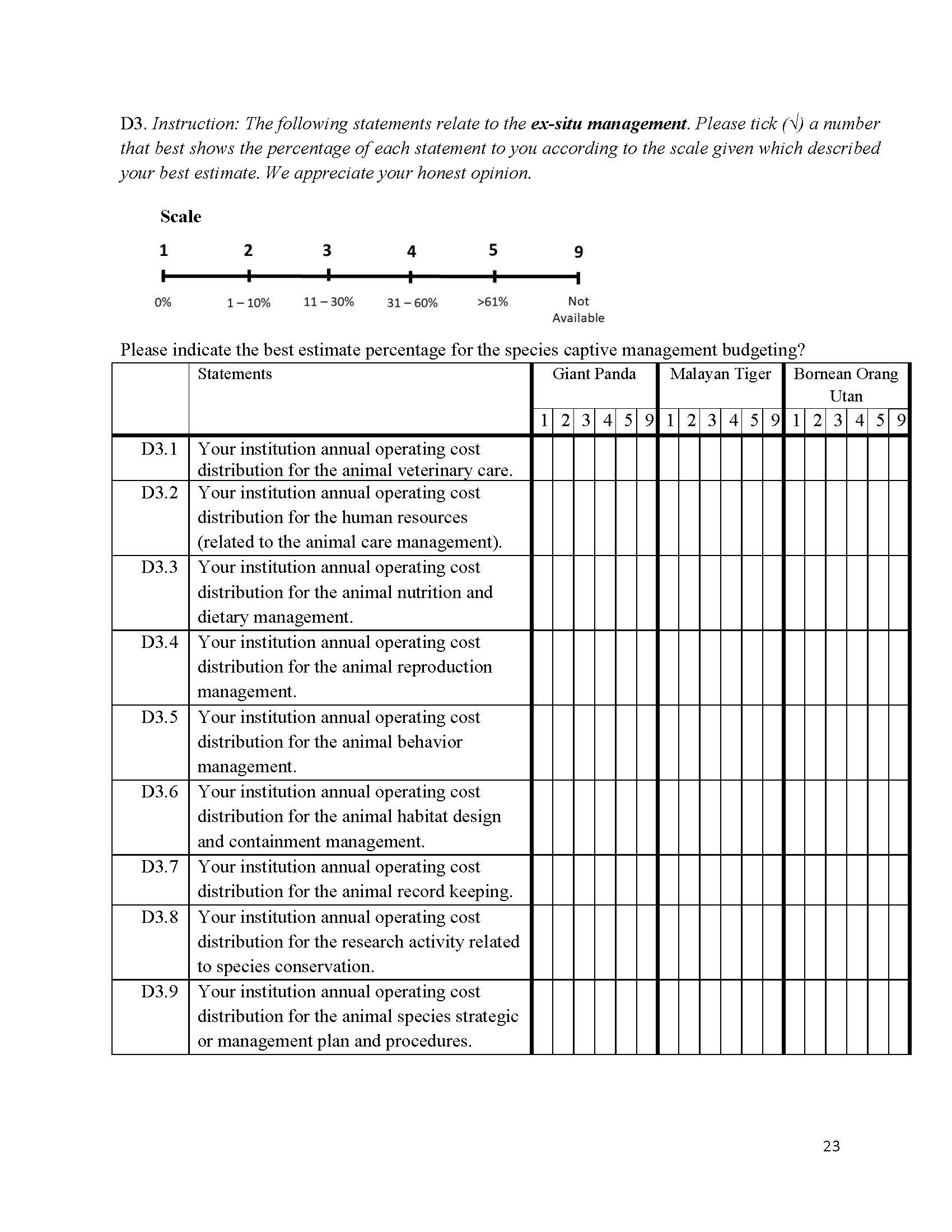** |
| **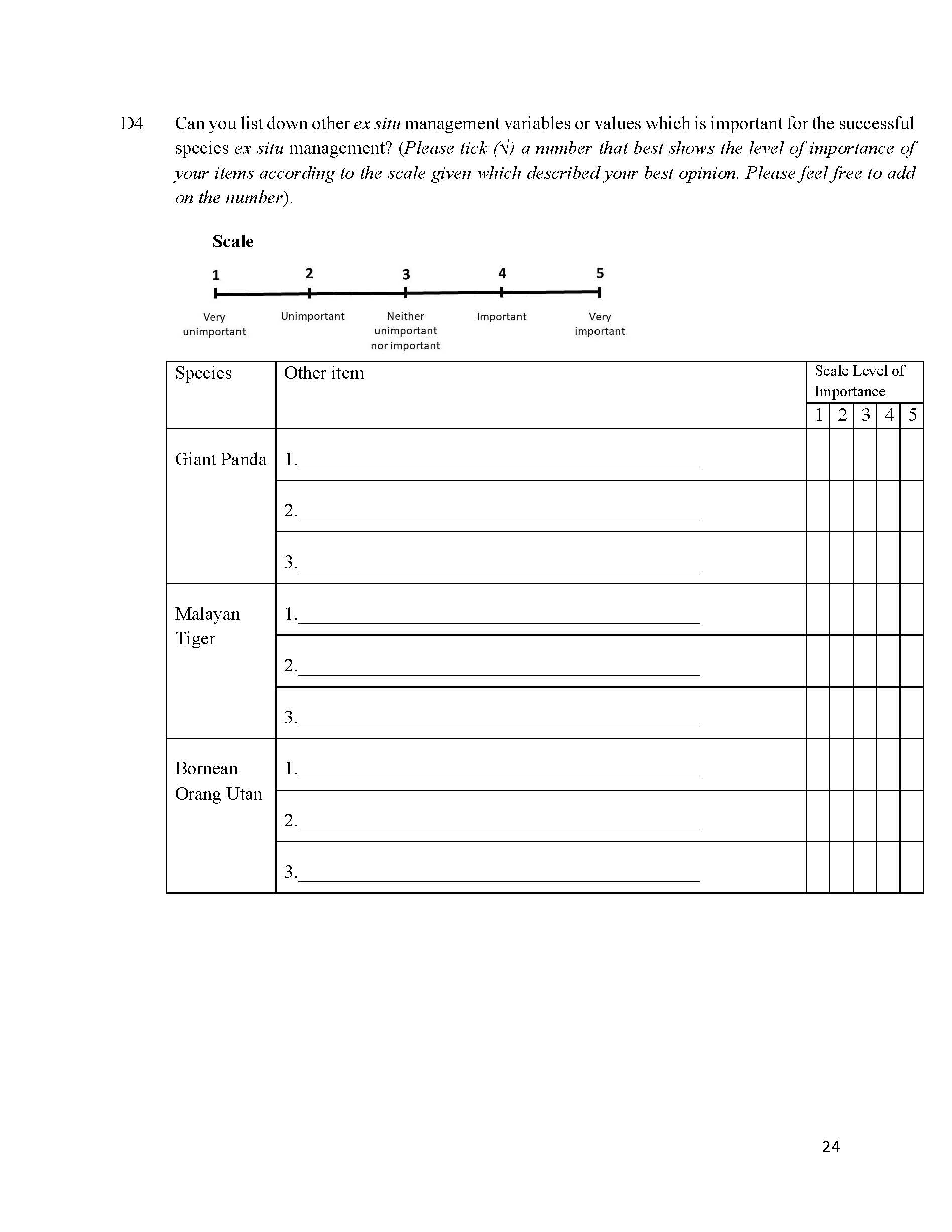** |
| **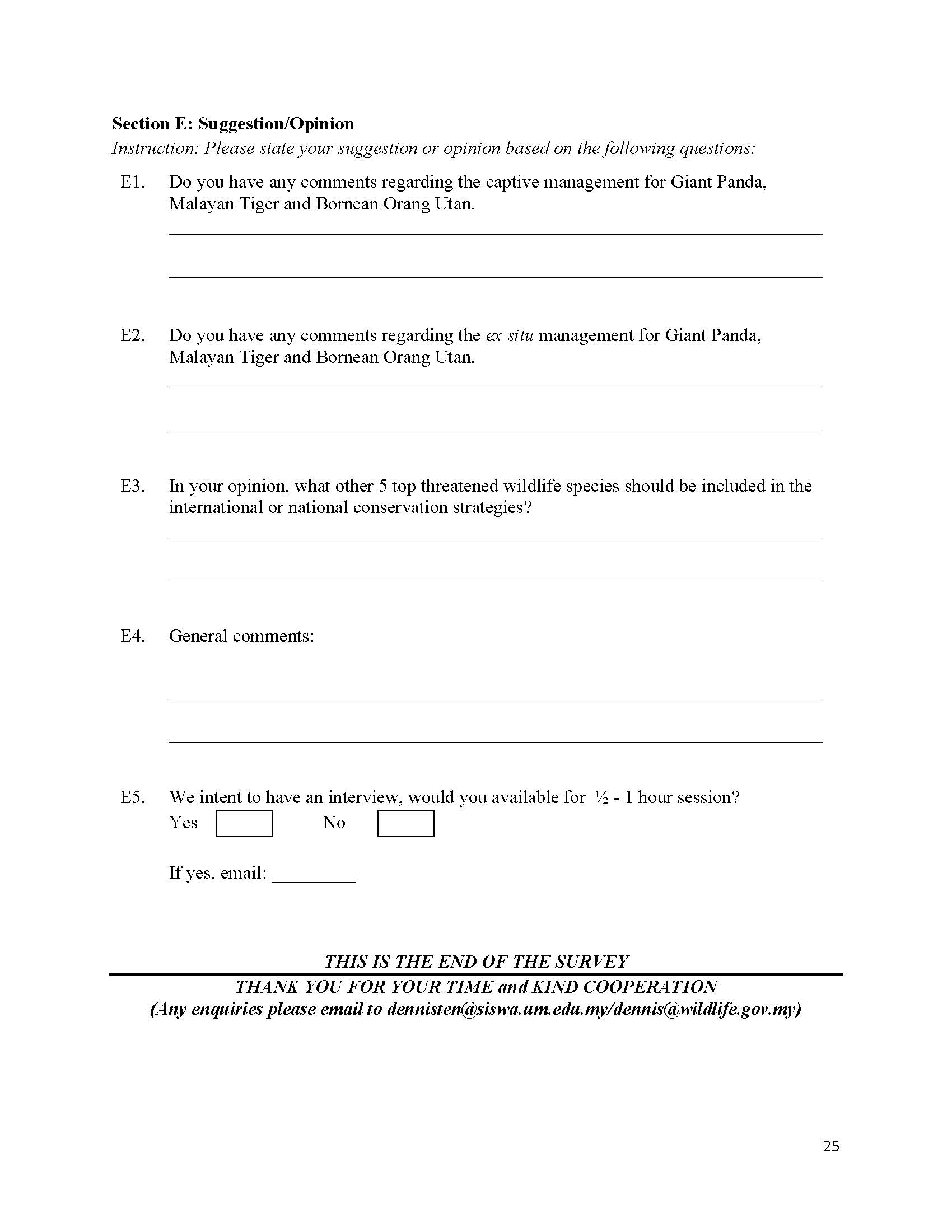** |

Supplement: Supplementary file 1 [file animals-11-01032-s001.zip › supplementary/Appendix A_Questionnaire_Malayan Tiger Mgmt Strategy_Conflict_AnimalsFormat_13022021.docx]
